# Island time and the interplay between ecology and evolution in species diversification

**DOI:** 10.1111/eva.12302

**Published:** 2015-11-17

**Authors:** Rosemary G. Gillespie

**Affiliations:** ^1^ Department of Environmental Science, Policy, and ManagementUniversity of CaliforniaBerkeleyCAUSA

**Keywords:** adaptive radiation, biodiversity, chronosequence, ecomorph, Hawaiian Islands, Pacific, spiders

## Abstract

Research on the dynamics of biodiversity has progressed tremendously over recent years, although in two separate directions – ecological, to determine change over space at a given time, and evolutionary, to understand change over time. Integration of these approaches has remained elusive. Archipelagoes with a known geological chronology provide an opportunity to study ecological interactions over evolutionary time. Here, I focus on the Hawaiian archipelago and summarize the development of ecological and evolutionary research; I emphasize spiders because they have attributes allowing analysis of ecological affinities in concert with diversification. Within this framework, I highlight recent insights from the island chronosequence, in particular the importance of (i) selection and genetic drift in generating diversity; (ii) fusion and fission in fostering diversification; and (iii) variability upon which selection can act. Insights into biodiversity dynamics at the nexus of ecology and evolution are now achievable by integrating new tools, in particular (i) ecological metrics (interaction networks, maximum entropy inference) across the chronosequence to uncover community dynamics and (ii) genomic tools to understand contemporaneous microevolutionary change. The work can inform applications of invasion and restoration ecology by elucidating the importance of changes in abundances, interaction strengths, and rates of evolutionary response in shaping biodiversity.

## Introduction

A grand challenge in understanding the origins of biodiversity is to ‘disentangle the influence of evolutionary and historical processes operating at larger spatiotemporal scales from ecological processes operating at smaller scales’ (Lessard et al. 2012). What makes this difficult is that ecological and evolutionary processes form a continuum and, while we can observe and test local ecological phenomena, we must usually infer evolutionary processes from current observations, often at larger spatial and temporal scales. Efforts to reconcile the interaction of ecological and evolutionary processes have largely adopted one of two approaches. The first makes maximal use of extensive sets of spatial data for broad comparative studies (Chase and Myers 2011; Belmaker and Jetz 2012); these studies use sophisticated approaches, with clustering analysis, network modularity analysis, and assemblage dispersion fields to define regional species pools (Carstensen et al. 2013). The far‐reaching scope of these studies has added immensely to our understanding of how regional large‐scale processes contribute to diversity at the local scale. A second approach uses detailed phylogenetic hypotheses across entire lineages to provide insights into change over evolutionary time. With the increasing availability of data across the diversity of life and broad spatial scales, this second framework can be coupled with data on current ecological traits and patterns of richness (Wiens et al. 2011; Anacker and Harrison 2012) to provide tests of how the interplay between ecological and evolutionary processes has shaped present‐day biodiversity (Graham et al. 2014). A missing element in both approaches is an understanding of how short‐term ecological processes, such as colonization and ecological fitting, can together give rise to larger and longer term processes of adaptation and diversification. In particular, understanding how community‐level ‘ecological’ processes play out into longer term ‘evolutionary’ processes is a black box in the understanding of biodiversity dynamics. Thus, a fundamental goal in biodiversity research is to add a dynamic framework to community ecology, which tends to view species as a fairly static pool (Mittelbach and Schemske 2015); this approach would allow a much needed understanding of the ecological/evolutionary interplay involved in the formation of biodiversity.

### Ecological insights into processes shaping species diversity

Until recently, ecological approaches to understanding parameters that dictate species composition, diversity, and community stability at a site primarily used one of two contrasting approaches. First, growing out of classic deterministic community ecology theory, manipulations of model vignette communities (with manageable species subsets in simplified mesocosms) or laboratory systems, coupled with simple dynamic theory, allowed tests for alternative mechanisms of local community interactions, such as predation or competition as limits to local diversity (e.g. Huffaker 1958; Paine 1966; Wilbur 1997; May 2001; Steiner et al. 2006). The main limitation of this approach is whether the results are relevant to more complex natural systems. In contrast, comparative approaches have applied statistical analysis of species composition of whole communities along abiotic gradients or time series to infer processes responsible for patterns of diversity (e.g. Pianka 1966; Rohde 1992). The main limitation here is that many hypotheses might explain similar patterns, making inferences on causation difficult (Palmer 1994).

From a theoretical perspective, exciting progress in understanding community structure comes from mechanistic models that can successfully predict strong central tendencies in quantitative food web patterns (Dunne 2006; Williams and Martinez 2008) and the effects of species loss and other dynamic population and community‐level properties (Berlow et al. 2009; Romanuk et al. 2009). The development of unified theories (neutral, continuum, metapopulation, fractal, clustered Poisson, MaxEnt) that establish a common set of rules to explain processes previously thought to be distinct has added richly to the understanding of biodiversity (Hubbell 2001; McGill 2010). One particularly powerful (but currently entirely spatial) theory, derived from maximum information entropy is the maximum entropy theory of ecology (METE; Harte 2011; Harte and Newman 2014), which provides quantitative ways to assess steady state and hence identify when a particular ecosystem is exhibiting unusual characteristics in common metrics, such as the species‐area relationship, species abundance distributions, spatial aggregation patterns (Brown 1995; Harte 2011), the distribution of metabolic rates over individuals in a community (Harte et al. 2009; Harte 2011), the inverse power‐law relation between abundance and body size (White et al. 2007), and the distribution of linkages across species in a trophic network (Williams 2010). Lacking to date is progress advancing these theories from the static to the dynamic so as to understand how variables change during community assembly, including effects of invasion and extinction.

### Difficulty of extrapolating ecological insights over evolutionary time

Adaptation and diversification are frequently studied independently from analyses of community assembly and structure, although there is increasing interest in linking the two, for example, in models of climate envelopes (Sutherst et al. 2007) and food webs (Loeuille and Loreau 2005; Johnson and Stinchcombe 2007). Attempts to assess the role of ecological processes in population differentiation and speciation have been limited due to the difficulty of making observations over evolutionary time, coupled with the complexity of most natural systems. However, much progress has been made through detailed studies of recent divergence (Schluter 2003, 2009) and associated micro‐evolutionary change (e.g. Roesti et al. 2014), although even in these studies, it is difficult to assess how short‐term effects of ecological interactions may translate into species formation (Losos 2010). Rapidly diversifying bacterial communities have provided microcosm systems allowing insights into the dynamics of diversification (Fukami et al. 2007; Meyer and Kassen 2007), but the challenge is to apply this knowledge more broadly (Gillespie and Emerson 2007). Each of these research angles, while highlighting the importance of integrating the fields of community ecology and evolutionary biology to understand processes involved in generating and maintaining biodiversity (Seehausen 2009; Palkovacs and Hendry 2010), also emphasizes the need for a well‐defined and simple system in order to measure and identify interactions (biotic and abiotic) and feedbacks.

To sum up, species diversity unfolds over evolutionary time in a highly complex manner that involves the entire community of organisms; the challenge is to find a study system that is simple enough to get a handle on all of the complexity, the history, the geography, and evolutionary adaptation. Islands – in particular remote oceanic islands that are discrete and self‐contained – provide a potential avenue for examining this complexity. And hotspot islands, which show a geological chronology, provide a potential opportunity for understanding the ecological–evolutionary continuum. The current paper aims to highlight this key role of remote islands in allowing insights into the processes underlying the formation of biodiversity at the intersection between ecology and evolution. I address: (i) How insular systems, in particular when chronologically arranged, provide opportunities to study the ecology/evolution continuum. Given this framework, one can bring in the players by highlighting; (ii) how different organisms, with a focus on Hawaiian spiders, have provided insights into diversification across the ecological chronosequence of the Hawaiian Islands. In particular, (iii) I examine key evolutionary insights that chronologically arranged islands are starting to provide, focusing on the roles of fusion/fission cycles, variability/plasticity, and the balance between natural selection and drift. Building on this, the culminating step is to consider (iv) how the Hawaiian Island chronology can be used to understand processes at the nexus between ecology and evolution. While focused on the Hawaiian Islands, I finish with a discussion of (v) parallel systems that might potentially allow similar inferences, and (vi) the conservation and related applied insights that can be gained from understanding biodiversity dynamics at the intersection between ecology and evolution.

## How can islands be used to understand eco‐evolutionary dynamics

### Natural selection and change through time

Insular systems, including islands and lakes, have played a fundamental role in providing insights into the operation of biological evolution (Gillespie and Baldwin 2009): The Galapagos islands in particular played a key role in the development of Charles Darwin's theory of evolution by means of natural selection (Grant and Grant 2008). Likewise, Alfred Russell Wallace contemporaneously developed similar theories based on studies in the Spice Islands of Indonesia (Severin 1997). More recently, the closed nature of insular systems, their relative simplicity, and often replicated pattern within a known temporal (geological or climatological) framework, has allowed them to serve as microcosms for understanding fundamental processes in evolution (Warren et al. 2015).

### Spatial ecology, theory, and applications

Islands have also served as a foundation for the development of key concepts in spatial ecology and biogeography. Perhaps the most important and influential theory was the equilibrium theory of island biogeography (ETIB) (MacArthur and Wilson 1967) that relates species numbers and area (*S* = *cA* ^*z*^) based on the premise that species diversity on an island is a balance between immigration and extinction, immigration decreasing with increasing distance from a mainland source, and extinction decreasing with increasing island size. The equilibrium theory marked a turning point in biogeography (Losos and Ricklefs 2009). Moreover, it has been applied to a vast diversity of insular systems, most notably to examine conservation implications of fragmentation on species diversity (Triantis and Bhagwat 2011).

Other key constructs in ecology have been based on island‐like systems. In particular, the Unified Neutral Theory of Biodiversity (Hubbell 2001) built on the ETIB to provide a general understanding of the diversity and relative abundance of species in ecological communities. Likewise, the METE, developed to describe the abundance, distribution, and energetics of species in a community, makes extensive use of island‐like habitats (Harte 2011; Harte and Newman 2014).

**Figure 1:**
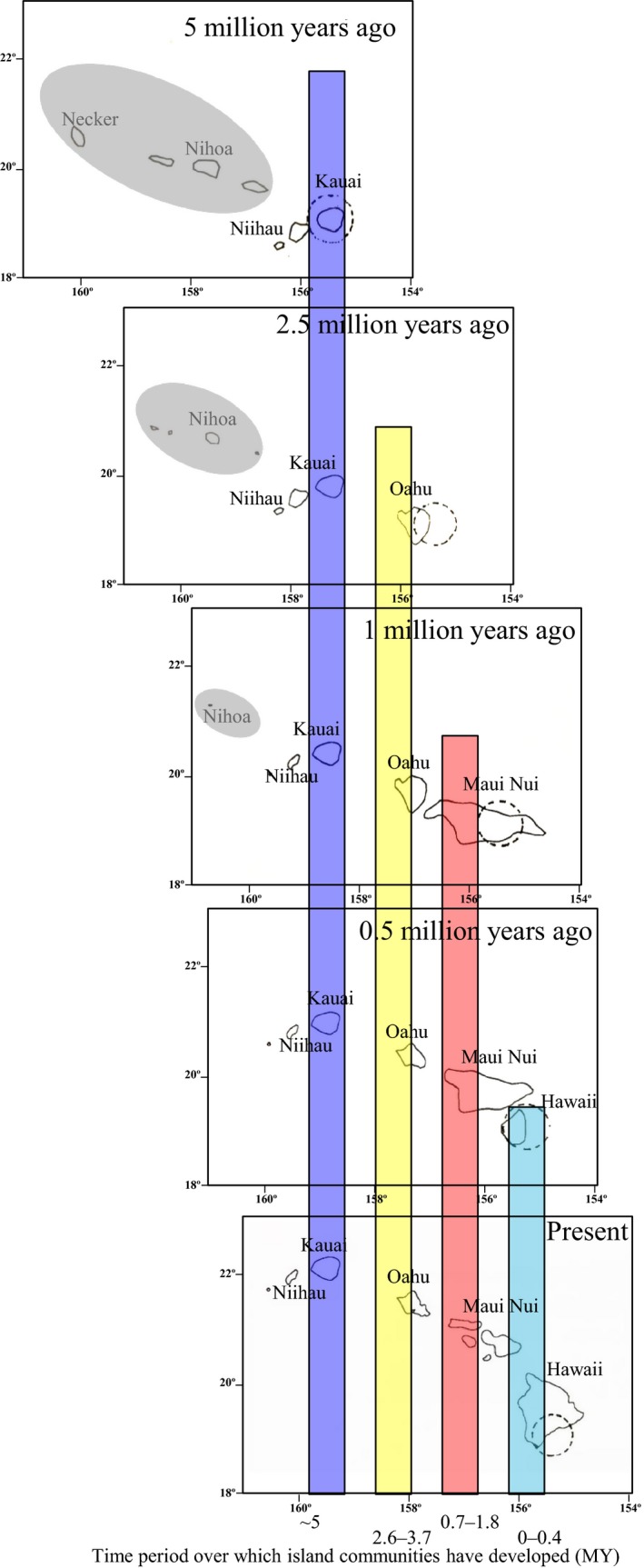
Map of the Hawaiian Islands at different time periods showing the age of the different island communities (Ma). Dashed circles indicate location of the volcanic hotspot. Colored bars indicate the period over which an island has existed. Gray circle around the northwest Hawaiian Islands in the top three panels indicate islands that generally do not form part of the biological chronosequence. These islands were small, low, and far apart when Kauai first emerged; thus, most taxa represented in the islands today originated from outside the archipelago rather than from the older islands, many colonizing Kauai at approximately 5 Ma (Price and Clague 2002). Map adapted from Carson and Clague (1995; Fig. 2.2).

Thus, islands have played a dual and key role in two very different research areas, evolutionary and ecological. However, despite the parallels between the basic genetic and ecological processes underlying biodiversity (Vellend 2010), integration between the explicitly temporal theories of evolutionary change and explicitly spatial theories of ecological change has proved extremely difficult to reconcile. The potential role of the Hawaiian Islands (Fig. 1) in providing insights on the interplay between ecology and evolution was first recognized over a century ago through the pioneering work of RCL Perkins (1913) (Box 1), although the sporadic nature of subsequent work meant that many of these earlier insights were largely overlooked until more recently (Grant 2000).

Box 1 **Setting the stage – early research in Hawaiian evolutionary biology and geology**

The multiple examples of adaptive radiation in the Hawaiian Islands led to some key early insights beginning with the pioneering discoveries of Perkins (1913), who worked in the islands from 1892 until 1902, spending extended periods collecting in the cold and wet mountainous areas at remote camps; these collections resulted in the publication of the *Fauna Hawaiiensis*, in three volumes (Perkins 1903). Key insights were published in the *Introduction* (Perkins 1913) where Perkins argued that species arose through a series of events involving divergence in geographical isolation followed by speciation, with competition increasing as new species were added (Grant 2000). Perhaps more important than his far‐sighted awareness of the evolutionary process, Perkins’ many years of field‐based research focused on multiple groups of organisms, allowed him to develop a thorough understanding of the ecological underpinnings of adaptive radiation (Evenhuis 2007). Thus, he noted the diversity of Hawaiian *Drosophila* and their ecological segregation based on larval breeding substrates (Kaneshiro 1997), “…Some of the species are quite conspicuous, and are readily attracted by the sap oozing from a broken limb of a tree, or from exudations caused by decay or disease. Very many breed in stems of trees or plants, which, when decaying, yield abundant moisture, such as those of the arborescent lobelia…” (Perkins 1913, p. 189). Perkins also noted the potential role of plasticity in fostering diversification, “It is not improbable that the plastic condition of the species in so many genera, and the extreme difficulty that exists in limiting the species, is really due to the slackness or absence of the agencies, by which natural selection works, the struggle for existence in the case of many of the island creatures having been much less severe than in a more populous and varied fauna”. (Perkins 1913, p. 64). The importance of plasticity in adaptive radiation is only now starting to gain recognition.

Geological understanding of remote hotspot islands in the early 20th century, while fairly rudimentary, allowed recognition that volcanoes and islands differed in age (Perkins 1913). Indeed, Perkins pointed out that certain volcanoes must have existed long before others, stating, “…the island of Hawaii, which is sometimes loosely stated to be the ‘youngest’ island… is a composite island, and its northern part is of very great age and existed long prior to the bulk of the island, and coincidently with the oldest of the other islands of the group”. Clearly, the detailed chronology of the islands was not well understood, although island age was implicit in the ancient mythological story of Pele and Hi'iaka, two sisters from Tahiti, who were said to have come to the northwestern Hawaiian Islands from Tahiti; Pele subsequently travelled down the island chain until finding her current home in the active volcano of Kilauea, on Hawai'i Island (Lund 1999).

Deeper understanding of the geological history of the islands did not come until much later. The idea that the Hawaiian and other volcanic island chains may have formed due to the movement of a plate over a stationary ‘hotspot’ in the mantle was proposed by Wilson (1963) who suggested that the islands arose, “like a series of bubbles arising from a point beneath the island of Hawaii” (Fig. 1). This hypothesis eliminated an apparent inconsistency to the plate‐tectonics theory – the occurrence of active volcanoes that were many thousands of kilometers from the nearest plate boundary. This idea was soon to be embraced by evolutionary biologists.

## The study system – value of Hawaiian spiders for studies integrating ecology and evolution

Adaptive radiations are characterized by rapid diversification that results in clades with broad phenotypic diversity (Schluter 2000), but without comparable levels of genetic divergence (Givnish and Sytsma 1997). The Hawaiian Islands are well known for spectacular examples of adaptive radiation, although understanding of the extent and nature of diversification has been slow: Only 1% of the known terrestrial animals in Hawaii are vertebrates (almost all birds) (Eldredge and Evenhuis 2003), and a broad understanding of eco‐evolutionary processes has been hindered by incomplete knowledge of the 99% (mostly arthropods) (Box 2).

Box 2 **Hawaiian terrestrial arthropods, in particular spiders – taxonomic history**

Taxonomic understanding of the Hawaiian fauna (notably arthropods) has progressed in spurts (Howarth and Gagné 2012), although remarkably few spurts. Following the *Fauna Hawaiiensis* (Perkins 1903), the next major undertaking was by E.C. Zimmerman who set out to develop keys and descriptions that would allow identification of all Hawaiian insects (Zimmerman 1948). Zimmerman accomplished this ambitious feat largely single‐handed, with help only for the Diptera (Hardy 1965) although more recent volumes have been authored by others (Christiansen and Bellinger 1992; Liebherr and Zimmerman 2000; Daly and Magnacca 2003). The *Insects of Hawaii* volumes and results of the *Drosophila* Project were instrumental in Hawaii being included as a site in the International Biological Programme (IBP) in 1970, serving to foster extensive biological – and especially entomological – research in the islands. By the 1990s, a checklist of the Hawaiian insects had gone from the 3245 listed in the *Fauna Hawaiiensis* to 7653 species, of which 4987 were endemic and by 2002 the tally included 8706 species of which 5366 were endemic (Nishida 2002). However, the 1980s saw a collapse in the funding situation for taxonomic research, with key institutions – the Bishop Museum in Honolulu being one of the first – falling on hard times. Thus, just when the importance of the arthropod fauna in Hawaii was finally being recognized, the rug was pulled out from underneath.

Among Hawaiian spiders, the *Fauna Hawaiiensis* collection of Perkins was studied in Paris by Simon (1900) who recognized the native spider species as belonging primarily to a handful of genera, most notably: *Tetragnatha* (Tetragnathidae), *Ariamnes* and *Theridion* (Theridiidae), *Labulla*, later transferred to *Orsonwelles* (Linyphiidae) (Hormiga 2002), *Cyclosa* (Araneidae); several genera of crab spiders, now all included in the genus *Mecaphesa* (Thomisidae) (Lehtinen and Marusik 2008), *Sandalodes*, later transferred to *Havaika* (Salticidae) (Prószyn'ski 2002), *Pagiopalus* and *Pedinopistha* (Philodromidae); and *Lycosa*,* Lycosella*, and *Syroloma* (Lycosidae). Over the next 90 years, the only major taxonomic work on Hawaiian spiders was by Suman (1970) on Thomisidae and Philodromidae, and Gertsch (1973) on two species of cave Lycosidae. Spiders were also not included in the *Insects of Hawaii* series and so had remained largely unknown. Thus, when I first arrived in the Hawaiian Islands in 1987, only eight species of a large adaptive radiation of *Tetragnatha* (Tetragnathidae) had been described from the islands (Simon 1900).

Since that time, I have described an additional 29 species of Hawaiian Tetragnatha (Gillespie 1991, 1992, 1994, 2002b, 2003a), with at least as many species left to describe. This species radiation encompasses forms representing a huge spectrum of colors, shapes, sizes, ecological affinities, and behaviors. Many species are web builders, with their shapes modified to allow concealment within specific microhabitats (Blackledge and Gillespie 2004); some species have modifications of the jaws, apparently to allow specialization on specific prey types (Gillespie 2005a); several groups have abandoned the characteristic web‐building behavior of the genus (Gillespie 1991, 2002b) with one entire clade of 16 species (the ‘spiny leg’ clade) having ‘lost’ web‐building behavior, with the concomitant development of long spines along the legs and adoption of a vagile, cursorial, predatory strategy. Additional taxonomic work on Hawaiian spiders over recent decades includes work on linyphiid sheet web spiders (Hormiga 2002), salticid jumping spiders (Prószyn'ski 2002, 2008), and thomisid crab spiders (Lehtinen and Murasik 2008), as well as the discovery of a new genus of barychelid brushed trapdoor spider on the atolls of the north west Hawaiian Islands (Churchill and Raven 1992).

Taxonomic understanding is integral to any insights into adaptive radiation. Thus, while highlighting the key role of molecular tools in facilitating the understanding of adaptive radiations (Givnish and Sytsma 1997), it is worth noting what has happened to the rate of new species descriptions. The situation in Hawaii reflects a global problem whereby understanding of the morphology and ecology of organisms has lagged far behind research using new genetic and genomic tools (Page 2013). With the increasing pressures of time, and escalating demands of permits and well‐cited publications, it is tempting for scientists to resort to DNA‐based approaches without consideration of taxonomy or natural history; however, the decreased emphasis on morphological and ecological characters also has resulted in a reduced understanding of the organism's place in the environment, and without such an understanding, one is effectively trying to build an edifice of knowledge without having paid attention to the foundation.

**Figure 2:**
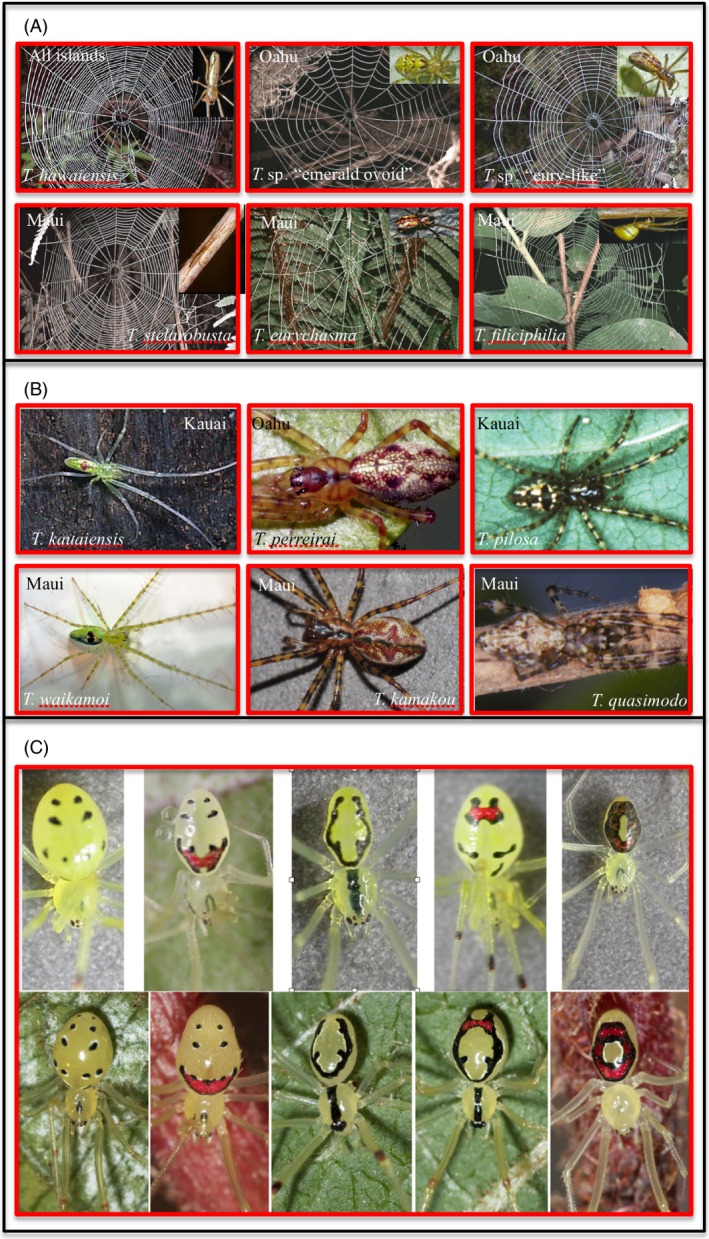
Convergence of similar ecological forms between species (A) and (B) and for diversity in form within a species (*Theridion grallator*, C) in Hawaiian spiders. Each red box indicates a single species. (A) Representatives of each of the primary web morphologies of the web‐building lineage of *Tetragnatha* spiders where different web types on the same island are more closely related than the same web type on different islands; however, the species that build the webs (inset photographs) show no eco‐morphological convergence (Blackledge and Gillespie 2004). (B) Three of four known ecomorphs of the spiny leg on older (Kauai, Oahu) and younger (Maui) islands (Gillespie 2004); the taxa shown from different ecomorphs on the same island are more closely related to each other than to the same ecomorph on different islands. (C) Happy face spiders, *Theridion grallator*, from Hawaii Island (top) and West Maui (bottom) – a set of corresponding color morphs found in a single population at one time. Photograph credits: (A) webs Todd Blackledge; insets R. Gillespie; (B) R. Gillespie except *Tetragnatha kamakou*, Darko Cotoras; (C) top row, G. Oxford, bottom row R. Gillespie.

Spiders are particularly suitable study organisms for examining patterns of diversification at the intersection between ecology and evolution, in part because their ecology is readily apparent through their very tight habitat affinities, and also because the web (when present) provides information on the ecology of the organisms. While Hawaiian spiders received little attention until recently (Box 2), studies are now starting to reveal insights into their origins, diversity, and evolutionary histories (Fig. 2). Thus, considering modes and directions of long distance dispersal, the long‐jawed spider genus *Tetragnatha* appears to have colonized Hawaii twice from the American continent, most likely by ‘ballooning’ on air currents (Gillespie et al. 1994). Phylogenetic reconstructions of another lineage, the Theridiinae (Theridiidae), support independent colonization of the Hawaiian Islands by two different lineages, one from the Americas, while the other has a holarctic sister relationship (Arnedo et al. 2007). *Cyclosa* (Araneidae) and the endemic *Orsonwelles* (Linyphiidae) may also have progenitors in the Americas. Jumping spiders in the genus *Havaika* (Salticidae) appear to have colonized the different archipelagoes of eastern Polynesia (Marquesas, Hawaii) independently, again most likely through ballooning from the Americas (Arnedo and Gillespie 2006), and the same may be true for thomisid crab spiders (Garb and Gillespie 2009). Both lineages (jumping spiders and crab spiders) in the eastern Pacific show a tight affinity between taxa in Hawaii and the Marquesas; given the large distance (almost equal to mainland‐island) between these very tiny islands, this pattern has been used to suggest that dispersal, at least for thomisids, may have been mediated by birds (Garb and Gillespie 2006, 2009). Large‐bodied Mygalomorph spiders in Hawaii are limited to two species in the genus *Nihoa* (Barychelidae), restricted to atolls on the northwest Hawaiian chain (Churchill and Raven 1992). Thus, spiders show the entire range of possible long‐distance dispersal modes, ballooning taxa most likely having made use of aerial dispersal, and likely from the Americas; some evidence of transportation by means of vectors, presumably migratory birds; and the large mygalomorphs being potential candidates for ocean rafting due to their low propensity for ballooning behavior and affinity with rotting wood and vegetation that can serve as rafts (Gillespie et al. 2012).

Within the Hawaiian Islands, the tendency to follow the ‘progression rule’ and diversify following the geological appearance of islands (Funk and Wagner 1995) has been demonstrated in several groups of spiders. Jumping spiders (*Havaika*, Salticidae) are the only lineage of spiders known to date that do not show a progression down the island chain, which has been attributed to a relatively recent arrival in the islands (Arnedo and Gillespie 2006). Groups that are inferred to have arrived on the older islands and progressed to the younger islands include the genus *Orsonwelles* (Linyphiidae) (Hormiga et al. 2003) and the large radiation of *Tetragnatha* (Tetragnathidae) (Gillespie 2004) as well as in Hawaiian crab spiders (Thomisidae) (Garb and Gillespie 2009) and likely also *Ariamnes* (Theridiidae) stick spiders (see below). Thus, a spectrum of lineages has been part of the biological community for periods ranging from 5 Ma (Kauai) to those currently being assembled on Hawaii Island (Fig. 1).

The value of endemic Hawaiian spiders in eco‐evolutionary studies is their broad spectrum of ecological affinities, and correspondingly different patterns of species diversity, while most have evolved over similar timeframes across the 5 Myr island chronosequence. I now consider the insights that these differences can provide into a broader understanding of differentiation over time.

## Recent insights from the Hawaiian chronosequence

Detailed understanding of the geological evolution of the Hawaiian Islands allows one to read the island chronology as a sort of fossil record, to look at the timeline of species diversification and extinction, and how it differs between taxa (Fig. 1). Major developments over the last few decades, in particular the advent of molecular tools, coupled with an increased recognition of the diversity of Hawaiian organisms (Box 3) have paved the way to some important recent breakthroughs, of which I highlight three that provide avenues for further exploration of the process of differentiation.

Box 3 **Developing insights into Hawaiian evolutionary biology and geology (1960–1995)**

Toward the second half of the 19th century, with increasing knowledge of the geology of the islands and initial ideas as to how organisms might be diversifying within the landscape, the major roadblocks to unraveling the process of adaptive radiation were (i) lack of tools for inferring evolutionary history and (ii) incomplete knowledge of the organisms. Galvanized by taxonomic and systematic feats by Zimmerman (1958) and Hardy (1965), some of the earliest work resulted from the Hawaiian *Drosophila* Project, initially funded by the National Institute of Health in 1963. In the 1960s, notable pioneering work by Hampton Carson examined the mechanism through which the geology of the Hawaiian Islands fostered genetic and functional diversity. He used polytene chromosomal inversions to map the evolutionary history of species across the archipelago (Carson 1970), culminating in an extraordinarily detailed understanding of phylogenetic connections between taxa in the diverse picture winged clade (Carson 1983, 1987). Results from these studies clearly showed that most species on the younger islands have evolved from the older islands.

The late 1980s started to see a recognition of the potential value of the Hawaiian Islands as a focus for studies integrating evolution and ecology, with the publication of a special issue on the islands edited by Chris Simon (1987) in *Trends in Ecology and Evolution*. The subsequent years benefited tremendously from molecular approaches. For example, while early studies had relied on patterns of ploidy in plants (e.g. Gardner 1976) and of polytene chromosomes in *Drosophila* (Carson 1970), methods such as DNA‐DNA hybridization started to be used in birds (Sibley and Ahlquist 1982) and *Drosophila* (Triantaphyllidis and Richardson 1982), and allozyme electrophoresis both in plants (Helenurm and Ganders 1985; Lowrey and Crawford 1985; Witter and Carr 1988; Aradhya et al. 1993) and flies (Carson 1982).

The development of molecular tools, while having a huge impact on evolutionary studies everywhere, was particularly important in Hawaii. Here, the many cases of rapid and strongly ecologically driven adaptive radiations make traditional phylogenetic studies based on morphological characters difficult due to the tendency of morphological synapomorphies to be few and frequently dominated by convergence (Givnish and Sytsma 1997). The molecular approaches allowed independent assessment of phylogeny, and thus a framework with which to examine the evolution of morphological, ecological, behavioral, and physiological adaptations, and the circumstances under which they have allowed species’ proliferation. The first studies to employ sequencing approaches to examine the evolutionary history of Hawaiian terrestrial organisms not surprisingly used *Drosophila* (Desalle et al. 1986), but additional molecular studies in other groups started in the early 1990s, with work on *Dubautia* (Baldwin et al. 1990, 1991). It was during this period that a hitherto largely unknown radiation of spiders was re‐discovered, that of the long‐jawed orb weaving genus *Tetragnatha* (Croom et al. 1991; Gillespie et al. 1994; Holmes and Harvey 1994).

With the growing interest in Hawaiian evolutionary biology, in large part facilitated by the increasing availability of tools for determining phylogenetic relationships, Warren Wagner and Vicki Funk organized a symposium to examine the developments in Hawaiian biogeography, an effort that resulted in a now classic volume (Funk and Wagner 1995), which described multiple radiations, all analyzed with regards their tendency to show an evolutionary progression down the island chain (i.e. the progression rule). That the geological history is mirrored in patterns of diversification for many Hawaiian adaptive radiations, and gives rise to sequential bouts of speciation upon successively younger islands, has since been borne out in radiations of many taxonomic groups (Wagner and Funk 1995; Roderick and Gillespie 1998).

### Balance between natural and sexual selection and genetic drift

An important finding for the evolution of Hawaiian taxa is that the pattern of diversification differs markedly between lineages, presumably through differences in the balance between ecological isolation caused by natural selection and geographic isolation through genetic drift, although it can also be accelerated through sexual selection. The balance of these processes shapes the extent of adaptive radiation in each lineage. Courtship or sexual behavior appears to be particularly effective in promoting rapid divergence, leading to Hawaiian crickets in the genus *Laupala* having one of the fastest rates of speciation so far recorded in arthropods (Mendelson and Shaw 2005). Similarly, fast rates of diversification have been noted in Hawaiian *Drosophila* (Kambysellis et al. 1995), with key insights into the role of sexual selection in driving rapid speciation (Boake 2005). But, given that sexual selection tends to augment other selective pressures (Ritchie 2007), I focus here on the balance between natural selection and genetic drift which is well illustrated in spiders, with some Hawaiian lineages characterized by little ecological differentiation, others by extensive ecological differentiation. Three primary patterns can readily be distinguished:

1. ’Nonadaptive’ radiation (Fig. 3A). *Orsonwelles* (Linyphiidae), with 13 species across all the islands, is a classic example of a ‘nonadaptive radiation’ with all species having similar ecologies and little evidence of species co‐occurring with each other (Hormiga et al. 2003).

**Figure 3:**
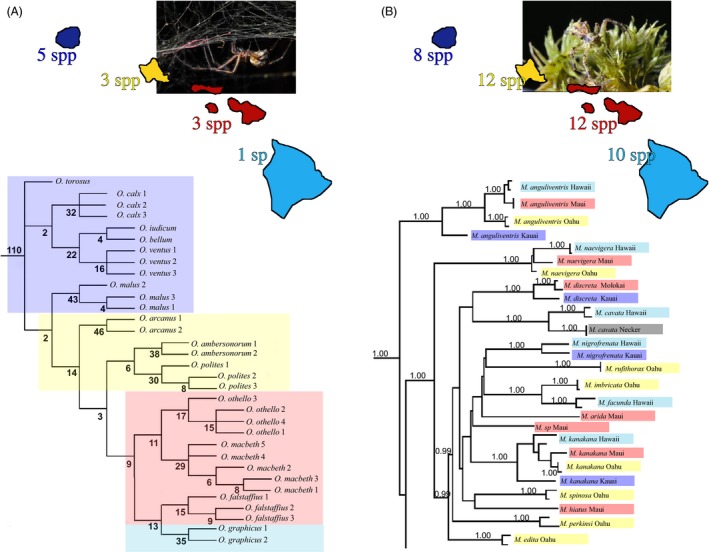
Phylogenetic relationships among radiations of: (A) *Orsonwelles* sheet web spiders, showing the progression down the island chain (Hormiga et al. 2003). Species numbers have increased with island age. Values below branches indicate Bremer support. (B) *Mecaphesa* crab spiders, showing early diversification into different ecologically defined taxa, and subsequent general progression down the island chain in each species; here, species numbers are high even on the youngest island and seem to level off quickly on the older islands (Garb and Gillespie 2009). Values above branches indicate posterior probabilities. Colors around taxa denote island. Inset photo credits A, G. Hormiga; B, J.E. Garb.
2. Adaptive radiation with ecological shifts limited to early in the radiation (Fig. 3B). In *Mecaphesa* crab spiders, it appears that ecological differentiation and niche shifts occurred largely on the oldest island of Kauai, which is not altogether surprising as isolation would have been extreme when Kauai was the only high island in the archipelago (Fig. 1; Price and Clague 2002). Subsequently, ecologically differentiated taxa appear to have colonized, largely independently, down the island chain, with multiple ecologically differentiated species co‐occurring at any one site (Garb and Gillespie 2009).
3. Adaptive radiation with repeated episodes of ecological differentiation (Figs 2 and 4). The genus *Tetragnatha* has undergone a particularly remarkable adaptive radiation in the Hawaiian Islands (Box 2), the best studied component being the ‘spiny leg’ clade of 17 species that has abandoned web‐spinning and adopted a wandering lifestyle (Gillespie 1991, 2002b). Members of the ‘spiny leg’ clade exhibit one of four ecomorphological forms or ‘ecomorphs’, readily distinguishable by their appearance. Ecomorphs are a common feature of adaptive radiations resulting from parallel evolution of suites of ecology‐associated morphological attributes across the landscape of the radiation (Gillespie 2013) and are well illustrated outside Hawaii by cichlid fish in Nicaraguan (Muschick et al. 2011) and African (Muschick et al. 2012) lakes, *Anolis* lizards in the Caribbean (Losos 2009), and sticklebacks in postglacial lakes (Schluter and Nagel 1995). Among Hawaiian *Tetragnatha* spiders, ecomorphs are characterized by their color – whether *Green*,* Maroon*,* Small Brown,* or *Large Brown* – and the substrates upon which they find refuge during the day (green leaves versus maroon mosses, brown twigs, or branches) (Gillespie 1991; Carter 2009), these characters also being associated with different feeding behaviors and leg spine morphologies (Binford 2001; Carter 2009; R. G. Gillespie, unpublished data). Given the exclusively nocturnal behavior of the spiders and their very limited visual capacity, diurnal predation is the most likely selective pressure responsible for the close color matching (Oxford and Gillespie 2001); the most likely predators are honeycreepers for which spiders can form an important component of the diet (Amadon 1950). Within the spider radiation, the lineage has largely followed the progression rule (Box 3); the most derived species are mainly on the youngest islands, and most species have closest relatives on the same island (Gillespie 1999, 2004). Ecomorphs have arisen partly through (i) *in situ* diversification producing closely related species of different ecomorph and (ii) between‐island colonization in which species pre‐adapted to each of the niches arrive from older islands and subsequently differentiate in allopatry without change in ecomorph (Fig. 4B).

**Figure 4:**
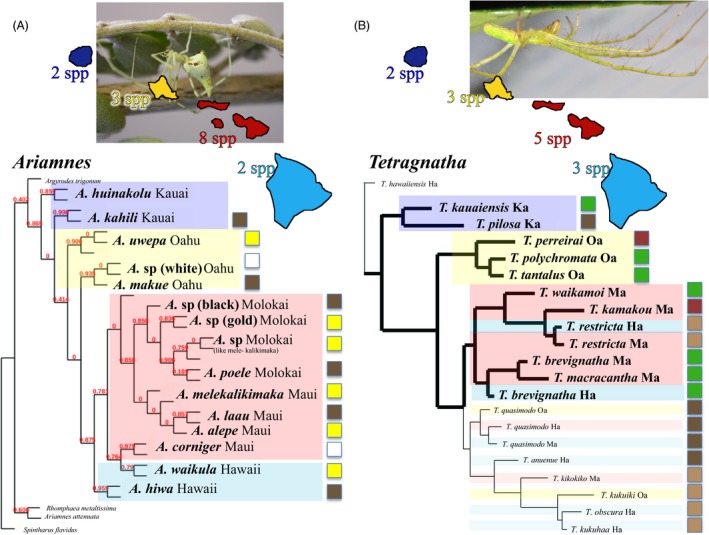
Phylogenetic relationships among radiations of: (A) Preliminary phylogeny of Ariamnes stick spiders based on mtDNA sequences, showing the progression down the island chain and repeated evolution of similar ecomorphs within islands. Values above branches indicate posterior probabilities. Species numbers increase early, and then decrease. (B) *Tetragnatha* long‐jawed spiders, also showing progression down the island chain and repeated evolution of similar ecomorphs (heavily shaded) as well as speciation between islands without any shift in ecomorph (subdued shading; the lower part of the phylogeny, Gillespie 2004). Colors around taxa represent island. Boxes to the right represent ecomorphological affinity.

Another clade within the Hawaiian *Tetragnatha* radiation comprises web builders. Here, ecomorphs can be defined by web form (Blackledge et al. 2003; Blackledge and Gillespie 2004), and here again, evidence suggests repeated evolution of ecomorphs. Interestingly, while web morphologies represent ecological associations, the morphology of the spiders themselves has changed in a manner unrelated to the web form (Fig. 2).

More recent work by Gillespie and Rivera (2007) has shown that *Ariamnes* stick spiders (Theridiidae) have undergone adaptive radiation much like that of the spiny leg *Tetragnatha*, with species exhibiting one of three ecomorphs – *Black*,* Gold*, or *White* – depending on which portion of the habitat they utilize (Gillespie 2004, 2013). Ecomorphs have arisen largely independently within islands, with convergence leading to similar sets between islands (Fig. 4A).

1. Convergence within single species. Surprisingly, convergence has been found within species, as well as between, as illustrated by the Hawaiian happy face spider *Theridion grallator* – a single species across the islands of Oahu, Molokai, Maui, and Hawaii. In any one population, this spider displays approximately eight distinct ‘color morphs’ (Gillespie and Tabashnik 1989; Gillespie and Oxford 1998; Oxford and Gillespie 2001) (Fig. 2C). Laboratory rearing experiments have indicated that this polymorphism is inherited in a Mendelian fashion, with the phenotypes exhibiting a genetic dominance hierarchy that reflects the extent of expressed pigmentation (Oxford and Gillespie 1996a). In virtually all populations, the polymorphism comprises a common cryptic yellow morph and numerous rarer, patterned morphs, and appears to be maintained by balancing selection (Gillespie and Oxford 1998). Remarkably, a fundamental change appears to have occurred in the mechanism of inheritance of the color polymorphism on the island of Hawaii (compared to Maui) with the most common morphs sex limited (unlike Maui) (Oxford and Gillespie 1996b, c). This suggests that the color polymorphism, or at least many of the rarer patterned morphs, may have been ‘reinvented’ on different island populations within the species (Croucher et al. 2012).

### Fragmentation and admixture

While a break in gene flow is necessary for adaptive differentiation, hybridization and genetic admixture resulting from previously separated populations coming back into contact with each other can be important in diversification: While sometimes leading to introgression among established species (Seehausen et al. 2008), genetic admixture has become increasingly recognized as a major contributor to adaptive variation and functional novelty (Seehausen et al. 2008). Several studies now demonstrate how the negative consequences of genetic founder effects may be offset if different colonization events result in multiple genotypes within the introduced population, highlighting the potential role of admixture among successively introduced populations in providing the genetic variation to allow adaptive evolution (Kolbe et al. 2007).

The importance of admixture in early diversification is not new to studies of Hawaiian organisms. Carlquist (1974) argued that natural hybridization can be a constructive force in the evolution of the waif flora: ‘As conditions change, gene flow among semi‐separate populations (and … taxonomic species) could maintain a high level of adaptability’. In Hawaiian silverswords, it has been shown that all crosses yield vigorous hybrids and the fertility of the F_1_ is dependent on the number of chromosome rearrangements differentiating parent species; introgression is theoretically possible between even the least interfertile species (Carr and Kyhos 1986). While such hybridization may lead to introgression and species ‘loss’, it may also have aided establishment or evolutionary success of a number of lineages, by elevating the genetic or genomic variation and potentially allowing for extensive recombination and expression of diverse phenotypes on which natural selection could act (Baldwin and Wagner 2010).

**Figure 5:**
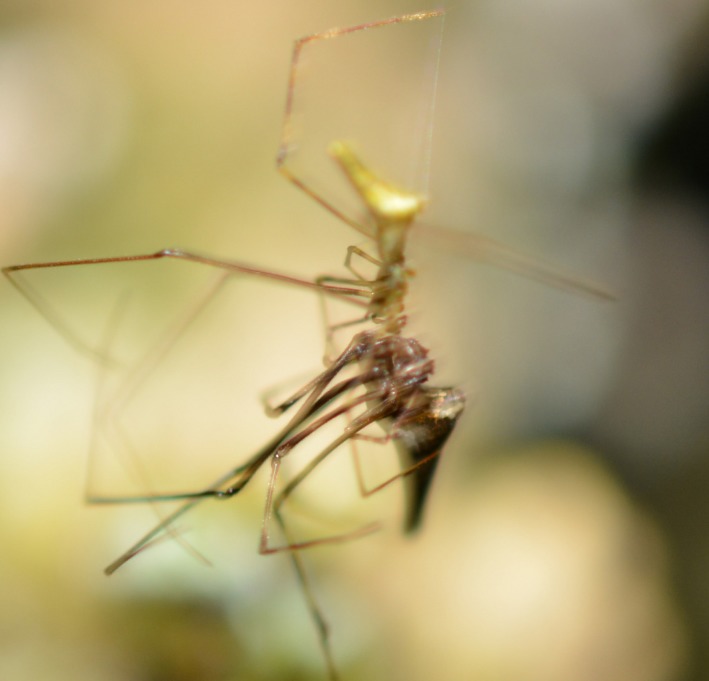
A field observation of a pair of *Ariamnes* from Waikamoi on the island of Maui. The upper individual is a male *Ariamnes alepeleke*, the lower a female *A*. laau. It appears that these spiders are mating, in which case it would be the first ever documentation of hybridization between two species of Hawaiian spiders, although it is unknown whether the mating was successful. Photograph by Susan Kennedy, March 2015.

Among arthropods, the role of hybridization is less clear. On one hand, the rapidity with which genetic incompatibilities can arise has been well documented, a recent study showing that parts of the genomes of *Drosophila* can diverge quickly, with strong detrimental fitness consequences of admixture between recently diverged taxa developing even at early stages of speciation (Fang et al. 2012). On the other hand, extensive hybridization and admixture are well known among Hawaiian arthropods with discordance of mtDNA and nuclear DNA phylogenies of Hawaiian *Drosophila* suggesting that genetic contact and hybridization between the ancestors may have played a role in shaping differentiation (DeSalle and Giddings 1986). Indeed, Carson et al. (1990) argued famously for the importance of the dynamic landscape of Hawaii created by lava flows, saying that, “…each species must continually recolonize from nearby areas or become extinct. This imposes a shifting mosaic population structure. …Since genetic adjustment depends on balanced, coadaptive polymorphisms, abundant polygenic recombinational genetic variability is retained [after a founder event]…” Thus, the repeated cycles of isolation and subsequent mixing of populations in new combinations (as a result of lava flows and subsequent vegetation regeneration) may provide an evolutionary crucible that can facilitate and potentially accelerate diversification (Carson et al. 1990). Here, the idea is that the negative consequences of genetic founder effects may be offset if different colonization events result in multiple genotypes within the introduced population; thus, admixture among successively colonizing populations could provide the genetic variation to allow adaptive evolution. Genomic approaches are now beginning to provide evidence of this phenomenon, although in other systems (Loh et al. 2013; Rius and Darling 2014). Interestingly, the arguments raised for Hawaii by Carson et al. (1990) have been echoed in the Galapagos, with Grant (Ahmed 2010) noting that the Galapagos finches “…exist in a kind of equilibrium state, dying and replenishing at a more‐or‐less equal rate over the long‐term. …This fusion/fission oscillation goes on a lot in biological systems. Hybrid finches .. mating pattern refuels the genetic pool of potential evolutionary responsiveness of each population…”. Evidence supporting the idea that lava flows can generate significant genetic differentiation has been shown among spiders in which lineages that have more restricted habitat preferences are subject to repeated episodes of isolation and fragmentation as a result of lava flows and vegetation succession (Vandergast et al. 2004). Moreover, it appears that the initial dynamic set up by the landscape translates over time into discrete lineages (Roderick et al. 2012): Given the same geographic distance, population divergence increases with time such that divergence is greater between the volcanoes of Maui Nui as compared to between the volcanoes on the Big Island. However, although admixture is expected to occur in the early diversification of spider lineages – with exciting recent observations possibly corroborating this suspicion (Fig. 5) – its role and extent has yet to be determined. Further work is needed to understand how genetic changes interact with a changing set of ecological interactions across a shifting mosaic of landscapes to promote species formation.

### Role of developmental variability

Polyphenism, in particular when associated with developmental variability and heterochrony, has been implicated in the early stages of adaptive radiation in diverse taxa (Price et al. 2003; Harmon et al. 2010; Muschick et al. 2011; O'Quin et al. 2011), suggesting that selection may act on some aspect of this variability to allow rapid differentiation. Indeed, polyphenism is associated with higher species richness in different clades of fish and amphibians (Pfennig et al. 2010). In terms of the mechanism by which diversification might happen, several studies, ranging from butterflies to fish, suggest that monophenic taxa are derived via fixation of a single phenotype from an ancestral taxon exhibiting polyphenism (West‐Eberhard 2003). Among sticklebacks, preliminary ontogenetic studies suggest that individuals of anadromous species are limnetic when young, becoming more benthic with age (West‐Eberhard 2005); if substantiated, this would imply that differentiation may involve alteration in the timing of expression of previously evolved adaptive traits related to these habitats (West‐Eberhard 2003). Likewise, selection acting on developmental polyphenism in beak shape among Galapagos finches seems to have played a role in differentiation (Abzhanov et al. 2004; Grant et al. 2006); and one of the basal African cichlid species appears to have undergone developmental shifts in genes associated with photoreceptor sensitivity, more derived taxa having evolved through repeated parallel evolution of opsin genes that are sensitive to either short‐ or middle‐wavelengths (O'Quin et al. 2011). Perhaps the most intriguing aspect of these studies is not just that polyphenism is tied to adaptive radiation, but that it is also associated with parallel evolution of similar eco‐morphological attributes (Gillespie 2013). These studies lend support to the argument that replicate speciation may arise from ancestral variability, perhaps as an expression of polyphenism rather than independent parallel evolution (West‐Eberhard 2003).

Island chronologies provide an ideal system in which to understand the evolutionary context within which developmental polyphenism might give rise to fixed differences, and how this might facilitate adaptive radiation: Among Hawaiian spiders, members of the ‘spiny leg’ clade show a gradation of developmentally linked color variability from older to younger islands (Brewer et al. 2015). Two species on the oldest islands in the archipelago (*Tetragnatha kauaiensis* and *Tetragnatha polychromata*) exhibit intraspecific (and mostly within‐individual) variability, changing from one ecomorph to another as the spiders age; more derived species on the younger islands show much less intraspecific variability, any one species or individual displaying a single ecomorph. Moreover, by examining variation in transcriptomes, we found clear signatures of selection associated with both loss of the color‐changing phenotype as well as colonization of a new environment. The next step will be to compare taxa at different ‘time slices’ across this radiation to show exactly how developmental polyphenism might serve as an avenue for the repeated evolution of ecomorphs during adaptive radiation.

Thus, we can see some common processes associated with adaptive differentiation in Hawaii, in particular that both fragmentation and admixture as well as developmental heterochrony, may play a role in providing variability upon which selection can act. At the same time, taxa differ in the nature and extent of diversification, in particular whether this involves adaptive shifts, and if so how readily. Thus, across communities of different ages, the nature and extent of divergence and adaptation will differ greatly between the players. Embracing these differences sets the stage for integrating the combined role of ecology and evolution in assembling communities over time.

## The Hawaiian Island chronology to integrate ecology and evolution

Ecological work in the terrestrial Hawaiian Islands has emphasized several key themes: (i) conservation biology, to determine ecological affinities of rare and endangered taxa (e.g. Vanderwerf 2012; Chau et al. 2013) or habitats (Howarth 1987); (ii) invasion biology to determine the impact of invasive species on natives (Vitousek et al. 1987; Cole et al. 1992; Krushelnycky and Gillespie 2008, 2010); and (iii) the ecological context of diversification (Kambysellis et al. 1995; Sakai et al. 1995; O'Grady et al. 2011; Goodman et al. 2012).

The use of the islands in providing a chronology for ecological studies and hence an opportunity to place research that is implicitly spatial, within a dynamic and temporal framework – has been used rather little. The primary exception is in ecosystem approaches to understand changes in soil and vegetation across the island chronology (Vitousek 2004), together with successional phenomena in vegetation dynamics focusing in particular on the dominant canopy tree *Metrosideros polymorpha* (Mueller‐Dombois 1987). An intriguing result from this work is that, along this gradient of some 4 million years of ecosystem development, nutrient availability and productivity peak at intermediate ages on the youngest island and begin to decline on the next older island and collapse on the oldest island. Nitrogen is most limited early on, with leaching of phosphorus from the parental material becoming most influential later on (Vitousek et al. 1995). Following the same gradient, Gruner (2007; Gruner et al. 2003) used whole biotic inventories of communities identified to morphospecies or functional groups, again finding that species richness peaks on islands of intermediate age (Gruner 2007).

**Figure 6:**
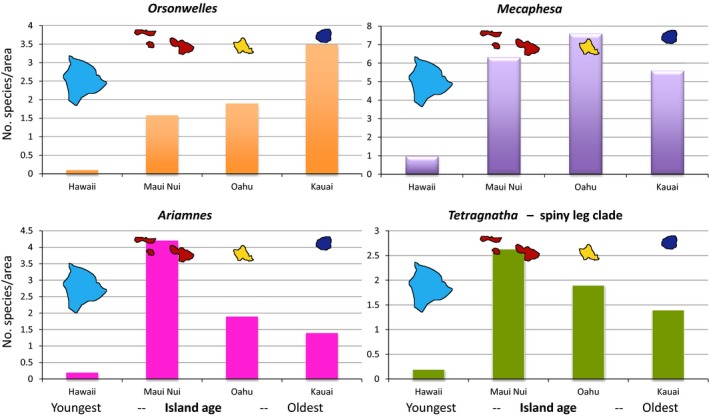
Species numbers per unit area for the major radiations of Hawaiian spiders. Values are shown for the major groups – *Orsonwelles* (Linyphiidae), *Mecaphesa* (Thomisidae), *Ariamnes* (Theridiidae), and *Tetragnatha* (Tetragnathidae) across the island chronology. Islands range in age from Kauai (oldest) to Hawaii (youngest) (see Fig. 1).

To understand how species diversity changes across the island chronology within specific lineages, Gillespie and Baldwin (2009) examined Hawaiian lineages that have been inferred to have been in the archipelago at least since the appearance of the current high islands (5 Ma) and showed that most species‐rich lineages of plants and animals reach their highest diversity (per unit area) on islands of intermediate age (Fig. 6). However, some lineages (in particular those that are less diverse) tend to show a steady increase in numbers. Two important implications from these results are as follows: (i) Patterns of species accumulation over evolutionary time in species‐rich lineages of remote islands are analogous to results from experimental tests of the ETIB, although species will clearly accumulate through speciation as well as immigration: Tests of the ETIB showed that immigration results in an overshoot in species number prior to a diversity decline and eventual stable state on islands close to the source of immigrants (Simberloff and Wilson 1970). (ii) Lineages appear to reach peak diversity at different rates and some may not have reached a stable state even on the oldest islands suggesting the possibility that, at least in some lineages, species numbers would continue to increase given enough time and persistence of terrestrial habitat. This raises the question as to whether there might be some predictability as to which lineages show which pattern. Interestingly, at least among the limited spider taxa we have studied to date (Figs 3 and 4), species diversity patterns across the Hawaiian chronosequence are associated with the extent and mechanism of adaptive radiation:

1. ’Nonadaptive’ radiation (*Orsonwelles*) – diversity increases linearly (perhaps even exponentially) with island age. This result is consistent with the knowledge that genetic distances between populations tends to increase with island age (Roderick et al. 2012), although it is interesting that a steady state appears not to have been reached, even on the oldest island of Kauai.
2. Adaptive radiation with ecological shifts limited to early in the radiation (*Mecaphesa* crab spiders) (Garb and Gillespie 2009) – diversity rises quickly, presumably because most ecological forms immigrate quickly to the new land mass from an older island.
3. Adaptive radiation with repeated episodes of ecological differentiation (*Ariamnes* and *Tetragnatha*). In these lineages, although initial divergence involves allopatry (Gillespie 2005b), speciation is frequently accompanied by sister taxa rapidly coming together in sympatry. It appears that, in the Hawaiian spider lineages that show this pattern of diversification, species numbers tend to increase rapidly on the younger islands, and then drop off on the older islands.

Thus, studies that have used the island chronology to examine ecosystem and biodiversity dynamics have mostly revealed nonlinear patterns of change over time.

## Parallel systems – a range of insular chronosequences

A common concern in considering the Hawaiian Island chronosequence as a system within which to examine the intersection between ecology and evolution is that it represents a sample size of one! So the question is, Are there other systems that can be used to generate the same kinds of data and test the same hypotheses?

Certainly, there are other hotspot archipelagoes. In the south Pacific, lineages occurring on increasingly isolated islands to the east tend to be a subset of those to the west (Gressitt 1982; Gillespie et al. 2008a). The Society Islands range from the oldest island of Maupiti at 4.3 Ma to the largest and youngest island of Tahiti at 2.0–0.5 Ma (Clouard and Bonneville 2005). One of the best studied genera of arthropods in the Society Islands is that of *Simulium* blackflies in which all species known today appear to have arisen on the youngest island of Tahiti (Joy and Conn 2001; Craig 2003). In long‐jawed spiders (*Tetragnatha*, Tetragnathidae) (Gillespie 2003c), the three known species represent two independent colonizations to the islands (Gillespie 2002a). The Marquesas, another hotspot archipelago in French Polynesia, ranging from Nuku Hiva, the oldest of the current high islands at 3.7 Ma, to Fatu Hiva the youngest at 1.8 Ma (Clouard and Bonneville 2005). Here, perhaps the best known radiation is that of the bird genus *Pomarea* (Monarchidae) which appears to have differentiated sequentially according to island appearance (Cibois et al. 2004). Among spiders, the genus *Tetragnatha* has undergone a small radiation (Gillespie 2003b), but the diversity is nothing close to that of the Hawaiian Islands. The islands of the Austral Archipelago, a hotspot south of the Societies, are sequentially ordered by increasing age, 4.5–12.2 Ma (Clouard and Bonneville 2005), although with secondary volcanic activity beneath the older islands. Among independent lineages of spiders, similar stories are emerging of sequential colonization of islands, with large genetic distances between island populations (Garb and Gillespie 2006; Gillespie et al. 2008a). Thus, the archipelagoes of the South Pacific show some striking examples of a ‘progression rule’, but this has been accompanied by little diversification (at least for spider lineages) in the Austral Islands, and rather little in the Marquesas or Societies – findings that should not be surprising given the very small size of these islands.

The Caribbean islands, although not entirely chronologically arranged, provide a system within which similar sets of ecological forms have evolved largely independently, together with some unique forms, as has been well documented in *Anolis* lizards (Langerhans et al. 2006; Losos 2009). Moreover, island age plays a role in species richness and associated island‐specific limits on total diversification (Rabosky and Glor 2010). However, the directionality of diversification across the islands of the Caribbean is unclear, with much debate on the role of vicariance versus dispersal in shaping the Caribbean biota and the importance of a hypothesized Greater Antilles‐Aves Ridge landbridge (‘GAARlandia’) between South America and the Greater Antilles during the Eocene Oligocene transition (Iturralde‐Vinent and MacPhee 1999). Following up on previous work across multiple organisms, studies on spiders in the genera *Selenops* (Selenopidae) (Crews and Gillespie 2010) and *Loxosceles* (Sicariidae: Loxoscleles) (Binford et al. 2008) have supported the GAARlandia hypothesis in the colonization of the islands from South America.

Beyond conventional islands, any habitat can serve as an island for a given organism depending on the extent to which it is restricted to a given habitat, its ability to cross the intervening matrix of inhospitable habitat, the distances involved, and its dispersal capability at a given time (Gillespie and Clague 2009). Thus, sky islands (McCormack et al. 2009), montane habitats (Porembski 2009; Rull 2009; Graham et al. 2014), and habitat fragments (Cayuela 2009) are often examined in the context of islands, the archipelagic nature coupled with repeated cycles of climate‐driven habitat expansion and contraction (Weir and Schluter 2004; Schoville et al. 2012) allowing some to serve as either ‘museums’ or ‘species pumps’ of diversity (Hutter et al. 2013). Although many of these systems do not show an easily identifiable chronology, a number do, and there is increasing information on a diversity of habitat chronosequences that might provide insights similar to those of the Hawaiian Islands (Wardle et al. 2004; Wardle 2006).

Perhaps the best known systems that parallel the chronosequence of the Hawaiian Islands are lakes of different ages within which evolutionary diversification progresses along the chronology. Among cichlids in the Great Rift Lakes, morphological and ecological convergence to similar form is strongly associated with the trophic niche of the organisms (Muschick et al. 2012), much like *Anolis* lizards (Losos 2009) and Hawaiian spiders (Gillespie 2004). In addition, the age of the lakes allows examination of patterns of co‐occurrence and can potentially be used to determine community‐level properties of the system (Brawand et al. 2014; Wagner et al. 2014) and how these change over time (Gillespie 2013). In the same way, other lake systems, in particular the alpine lake system in Switzerland, are starting to offer insights into biodiversity dynamics, using the natural spatio‐temporal pattern of the landscape (Brodersen and Seehausen 2014) and changes in ecological community characteristics over time (Melián et al. 2015).

## Dynamics of change and conservation implications

With the arrival of humans, the biological dynamics of isolated biological communities has changed completely, with immigration rates higher by orders of magnitude. While great strides have been taken to block the arrival of invasive species and mitigate their impacts, we have no models to show how native communities can accommodate, resist, or succumb to the new dynamic. To assess the future of the native biodiversity, it is necessary to understand the ecological and evolutionary dynamic of the nonindigenous species on these islands (Gillespie et al. 2008b). A major question is: Can we predict vulnerability or resilience based on community metrics?

Through ongoing research on community dynamics, colleagues and I are investigating (i) whether abundance distributions and trophic interactions change in a predictable way and (ii) how lineages have evolved *within* the age‐structured landscape of the youngest island within the Hawaiian chain using a set of arthropods from a range of trophic levels, habitat affinities, and species diversity (Rominger et al. 2015).

Efforts to predict invasion success have major limitations (Williamson 1996). Yet, models have examined possible general rules that govern invasion success within ecological networks that integrate models of food web structure and nonlinear dynamics (Williams and Martinez 2004). Similar rules are likely to govern ecological restoration success (Tylianakis et al. 2010). For species invasions, food web theory describes how each invader's trophic function is mediated by other species’ trophic activities and the structural topology of the invaded food web (Romanuk et al. 2009). This framework, together with METE kinds of approaches, provides an opportunity to make predictions about what types of communities are prone to invasion by what kind of invaders, and the new stability states of an invaded or restored community in terms of network structure and interaction strengths.

Communities of endemic Hawaiian species emerged when evolution outpaced immigration as a source of novel diversity; however, alien propagules now arrive faster than ever. The question then is, how are communities responding to the new dynamic? We hope our research will provide answers to the following: (i) What is the relative importance of priority, sequence, abundance, and interaction strengths in determining response to higher rates of immigration from non‐native taxa? (ii) Are non‐native species functional substitutes for native species? and (iii) Can we develop viable strategies for restoration?

## Conclusion

A major gap in understanding biodiversity dynamics is determining how small scale ‘ecological’ processes give rise to larger and longer term ‘evolutionary’ processes. Island chronologies provide one of the few systems in which to examine this dynamic – that is, how do ecological processes give way to evolutionary processes; they provide (i) a largely closed system and (ii) a well‐defined geological history such that younger islands or substrates hold the biologically youngest communities. My research has been using islands to understand eco‐evolutionary dynamics since 1987 (Box 4).

Box 4 **Personal perspective**

My message to those starting out in evolutionary biology, in particular women, is to love what you do with a passion – and do what you love with equal passion. I grew up in rural southwest Scotland, and always felt my calling in biology; I raised cats, mice … hundreds of each. I belonged to the British Mouse Fanciers Club and took immense pleasure in crossing individuals of different color, pattern, and hair length, to see what would be produced. However, my education through high school had a strong emphasis on areas such as deportment and ‘domestic’ science, lacking any obvious route to academia. The last 2 years of high school, spent in the north of Scotland, changed this trajectory and made it possible for me to go to Edinburgh University to study ecology. Graduating in 1980, I had experienced the excitement of research having already written a paper on spider feeding behavior. However, the Thatcher era in the UK saw universities being held accountable to market forces; a graduate degree was considered ‘procrastination’. My undergraduate advisor, Philip Ashmole, suggested an internship in the US to give me a taste for graduate research. A summer in the mountains of Colorado culminated in a circuitous bus ride across the US, via Knoxville where I met Susan Riechert, a leader in the field of behavioral ecology, in particular as it pertained to spiders. Susan opened the prospect of graduate school at the University of Tennessee. While exciting, the idea of spending the next few years in a country where we had not a single relative or friend, was a bit of a pill for my family. Four years later, having worked doggedly toward the notion of completing a PhD and returning to Scotland, it became obvious that I had to go where the research took me. Very serendipitously, I ended up in Hawaii, taking over a project on feeding behavior in happy face spiders from Sam Gon, who had just secured a position with The Nature Conservancy. Over the course of the ensuing fieldwork, and much help from Art Medeiros, I discovered the adaptive radiation of Hawaiian long‐jawed spiders. The finding of such an extraordinary and totally unknown phenomenon was intoxicating. The next few years were spent on minimal funds and maximal field time, but at the end I knew those spiders and their environment intimately. To quote Jim Brown ‘If you know one taxon and one region well, you begin to see patterns in the rest of the world’ (Adams 2007). Finding a partner, in my case George Roderick, who shared my enthusiasm for evolution and field biology, was an important step forward. And for me, children were a natural part of our lives, integrated into the research – how could they not be when research is so much part of me? Maybe they did not enjoy the relentless rain, or getting lost in the forest at night – but the experience became something that was unique to them, and they are proud of it. I think we all sometimes need to be reminded that we started in this field because we love biology and we are intrinsically curious; the hard work and dedication is almost an indulgence.

Islands have served as bedrock systems for the development of key theories in evolution, although only recently have molecular genetic and genomic tools allowed detailed insights into patterns of diversification. Spiders have proved particularly useful for this work, as ecomorphological attributes are readily measurable and provide insights into adaptive radiation that result from dynamic landscapes, polymorphism, and natural selection. Islands have also been integral in the development of ecological theory to generate predictive patterns of species abundance and diversity. Here again, Hawaiian spiders offer understanding as to how and why species diversity changes over evolutionary time. We now recognize the potential roles of the changing landscape in fostering cycles of genetic fusion and fission, and of developmental heterochrony in providing variability upon which selection can act. Moreover, we recognize that taxa differ fundamentally in rates of diversification and adaptive divergence. We also now have the tools to use islands to understand the intersection between shorter term ecological processes and longer term evolutionary processes in dictating the assembly of entire communities. This understanding of biodiversity dynamics is critical to the future of these unique communities – not only how diversity has been shaped in the past, but also how it will be expected to accommodate change in the future.

## References

[eva12302-bib-0001] Abzhanov, A. , M. Protas , B. R. Grant , P. R. Grant , and C. J. Tabin 2004 Bmp4 and morphological variation of beaks in Darwin's finches. Science 305:1462–1465. 15353802 10.1126/science.1098095

[eva12302-bib-0002] Adams, D. 2007 Profiles in biogeography: James H. Brown In Hortal J., ed. Profiles in Biogeography; International Biogeography Society. http://biogeography.blogspot.com/2007/11/dee-adams-profiles-in-biogeography.html (accessed on 9 September 2015).

[eva12302-bib-0003] Ahmed, F. 2010 Profile of Peter R. Grant. Proceedings of the National Academy of Sciences of the USA 107:5703–5705. 20339083 10.1073/pnas.1001348107 PMC2851900

[eva12302-bib-0004] Amadon, D. 1950 The Hawaiian honeycreepers (Ayes, Drepaniidae). Bulletin of the American Museum of Natural History 95:151–262.

[eva12302-bib-0005] Anacker, B. L. , and S. P. Harrison 2012 Historical and ecological controls on phylogenetic diversity in Californian plant communities. The American Naturalist 180:257–269. 10.1086/666650 22766935

[eva12302-bib-0006] Aradhya, K. M. , D. Mueller‐Dombois , and T. A. Ranker 1993 Genetic structure and differentiation in *Metrosideros polymorpha* (Myrtaceae) along altitudinal gradients in Maui, Hawaii. Genetical Research 61:159–170.

[eva12302-bib-0007] Arnedo, M. A. , and R. G. Gillespie 2006 Species diversification patterns in the Polynesian jumping spider genus *Havaika* Proszynski 2001 (Araneae, Salticidae). Molecular Phylogenetics and Evolution 41:472–495. 16837219 10.1016/j.ympev.2006.05.012

[eva12302-bib-0008] Arnedo, M. A. , I. Agnarsson , and R. G. Gillespie 2007 Molecular insights into the phylogenetic structure of the spider genus *Theridion* (Araneae, Theridiidae) and the origin of the Hawaiian *Theridion*‐like fauna. Zoologica Scripta 36:337–352.

[eva12302-bib-0009] Baldwin, B. G. , and W. L. Wagner 2010 Hawaiian angiosperm radiations of North American origin. Annals of Botany 105:849–879. 20382966 10.1093/aob/mcq052 PMC2876002

[eva12302-bib-0010] Baldwin, B. G. , D. W. Kyhos , and J. Dvorak 1990 Chloroplast DNA evolution and adaptive radiation in the Hawaiian silversword alliance (Asteraceae‐Madiinae). Annals of the Missouri Botanical Garden 77:96–109.

[eva12302-bib-0011] Baldwin, B. G. , D. W. Kyhos , J. Dvorak , and G. D. Carr 1991 Chloroplast DNA evidence for a North American origin of the Hawaiian silversword alliance (Asteraceae). Proceedings of the National Academy of Sciences of the USA 88:1840–1843. 11607157 10.1073/pnas.88.5.1840 PMC51121

[eva12302-bib-0012] Belmaker, J. , and W. Jetz 2012 Regional pools and environmental controls of vertebrate richness. The American Naturalist 179:512–523. 10.1086/664610 22437180

[eva12302-bib-0013] Berlow, E. L. , J. A. Dunne , N. D. Martinez , P. B. Starke , R. J. Williams , and U. Brose 2009 Simple prediction of interaction strengths in complex food webs. Proceedings of the National Academy of Sciences of the USA 106:187–191. 19114659 10.1073/pnas.0806823106 PMC2629248

[eva12302-bib-0014] Binford, G. J. 2001 Differences in venom composition between orb‐weaving and wandering Hawaiian *Tetragnatha* (Araneae). Biological Journal of the Linnean Society 74:581–595.

[eva12302-bib-0015] Binford, G. J. , M. S. Callahan , M. R. Bodner , M. R. Rynerson , P. B. Nunez , C. E. Ellison , and R. P. Duncan 2008 Phylogenetic relationships of *Loxosceles* and *Sicarius* spiders are consistent with Western Gondwanan vicariance. Molecular Phylogenetics and Evolution 49:538–553. 18755282 10.1016/j.ympev.2008.08.003

[eva12302-bib-0016] Blackledge, T. A. , and R. G. Gillespie 2004 Convergent evolution of behavior in an adaptive radiation of Hawaiian web‐building spiders. Proceedings of the National Academy of Sciences of the USA 101:16228–16233. 15520386 10.1073/pnas.0407395101 PMC528981

[eva12302-bib-0017] Blackledge, T. A. , G. J. Binford , and R. G. Gillespie 2003 Resource use within a community of Hawaiian spiders (Araneae: Tetragnathidae). Annales Zoologici Fennici 40:293–303.

[eva12302-bib-0018] Boake, C. R. B. 2005 Sexual selection and speciation in Hawaiian *Drosophila* . Behavior Genetics 35:297–303. 15864444 10.1007/s10519-005-3221-4

[eva12302-bib-0019] Brawand, D. , C. E. Wagner , Y. I. Li , M. Malinsky , I. Keller , S. Fan , O. Simakov et al. 2014 The genomic substrate for adaptive radiation in African cichlid fish. Nature 513:375–381. 25186727 10.1038/nature13726 PMC4353498

[eva12302-bib-0020] Brewer, M. S. , R. A. Carter , P. J. P. Croucher , and R. G. Gillespie 2015 Shifting habitats, morphology, and selective pressures: developmental polyphenism in an adaptive radiation of Hawaiian spiders. Evolution 69:162–178. 25403483 10.1111/evo.12563

[eva12302-bib-0021] Brodersen, J. , and O. Seehausen 2014 Why evolutionary biologists should get seriously involved in ecological monitoring and applied biodiversity assessment programs. Evolutionary Applications 7:968–983. 25553061 10.1111/eva.12215 PMC4231589

[eva12302-bib-0022] Brown, J. H. 1995 Macroecology. University of Chicago Press, Chicago, IL.

[eva12302-bib-0023] Carlquist, S. 1974 Island Biology, 660 pp. Columbia University Press, New York, NY and London. 581:5279.

[eva12302-bib-0024] Carr, G. D. , and D. W. Kyhos 1986 Adaptive radiation in the Hawaiian silversword alliance (Compositae‐Madiinae). II. Cytogenetics of artificial and natural hybrids. Evolution 40:959–976. 10.1111/j.1558-5646.1986.tb00565.x 28556216

[eva12302-bib-0025] Carson, H. L. 1970 Chromosome tracers of the origin of species. Science 168:1414–1418. 5445927 10.1126/science.168.3938.1414

[eva12302-bib-0026] Carson, H. L. 1982 Evolution of *Drosophila* on the newer Hawaiian volcanoes. Heredity 48:3–25. 7042651 10.1038/hdy.1982.2

[eva12302-bib-0027] Carson, H. L. 1983 Chromosomal sequences and interisland colonizations in Hawaiian *Drosophila* . Genetics 193:465–482. 17246115 10.1093/genetics/103.3.465 PMC1202034

[eva12302-bib-0028] Carson, H. L. 1987 Tracing ancestry with chromosomal sequences. Trends in Ecology & Evolution 2:203–207. 21227851 10.1016/0169-5347(87)90021-8

[eva12302-bib-0029] Carson, H. L. , and D. A. Clague 1995 Geology and biogeography of the Hawaiian Islands In Wagner W. L., and Funk V. A., eds. Hawaiian Biogeography: Evolution on a Hot Spot Archipelago, pp. 14–29. Smithsonian, Washington, DC.

[eva12302-bib-0030] Carson, H. L. , J. P. Lockwood , and E. M. Craddock 1990 Extinction and recolonization of local populations on a growing shield volcano. Proceedings of the National Academy of Sciences of the USA 87:7055–7057. 11607102 10.1073/pnas.87.18.7055 PMC54681

[eva12302-bib-0031] Carstensen, D. W. , J.‐P. Lessard , B. G. Holt , M. Krabbe Borregaard , and C. Rahbek 2013 Introducing the biogeographic species pool. Ecography 36:1310–1318.

[eva12302-bib-0032] Carter, R. A. 2009 Behavioral and morphological plasticity in an adaptive radiation of Hawaiian “Spiny Leg” Tetragnatha spiders (Araneae: Tetragnathidae). Doctoral Dissertation, Environmental Science, Policy and Management, University of California, Berkeley, CA, USA.

[eva12302-bib-0033] Cayuela, L. 2009 Fragmentation In Gillespie R. G., and Clague D., eds. Encyclopedia of Islands, pp. 328–330. University of California Press, Berkeley, CA.

[eva12302-bib-0034] Chase, J. M. , and J. A. Myers 2011 Disentangling the importance of ecological niches from stochastic processes across scales. Philosophical Transactions of the Royal Society of London B: Biological Sciences 366:2351–2363. 21768151 10.1098/rstb.2011.0063 PMC3130433

[eva12302-bib-0035] Chau, M. M. , W. R. Reyes , and T. A. Ranker 2013 Ecological factors influencing growth of the endangered Hawaiian fern *Marsilea villosa* (Marsileaceae) and implications for conservation management. American Journal of Botany 100:1532–1543. 23857737 10.3732/ajb.1200625

[eva12302-bib-0036] Christiansen, K. , and P. Bellinger 1992 Insects of Hawaii. Volume 15, Collembola. University of Hawai'i Press, Honolulu, viii + 445 pp.

[eva12302-bib-0037] Churchill, T. B. , and R. J. Raven 1992 Systematics of the intertidal trapdoor spider genus *Idioctis* (Mygalomorphae: Barychelidae) in the western Pacific with a new genus from the northeast. Memoirs of the Queensland Museum 32:9–30.

[eva12302-bib-0038] Cibois, A. , J.‐C. Thibault , and E. Pasquet 2004 Biogeography of eastern Polynesian monarchs (Pomarea): an endemic genus close to extinction. The Condor 106:837–851.

[eva12302-bib-0039] Clouard, V. , and A. Bonneville 2005 Ages of seamounts, islands, and plateaus on the Pacific plate In Foulger G. R., Natland J. H., Presnall D. C., and Anderson D. L., eds. Plates, Plumes, and Paradigms, pp. 71–90. Geological Society of America, Boulder CO.

[eva12302-bib-0040] Cole, F. R. , A. C. Medeiros , L. L. Loope , and W. W. Zuehlke 1992 Effects of the Argentine ant on arthropod fauna of Hawaiian high‐elevation shrubland. Ecology 73:1313–1322.

[eva12302-bib-0041] Craig, D. A. 2003 Geomorphology, development of running water habitats, and evolution of black flies on Polynesian islands. BioScience 53:1079–1093.

[eva12302-bib-0042] Crews, S. C. , and R. G. Gillespie 2010 Molecular systematics of *Selenops* spiders (Araneae: Selenopidae) from North and Central America: implications for Caribbean biogeography. Biological Journal of the Linnean Society 101:288–322.

[eva12302-bib-0043] Croom, H. B. , R. G. Gillespie , and S. R. Palumbi 1991 Mitochondrial DNA sequences coding for a portion of the RNA of the small ribosomal subunits of *Tetragnatha mandibulata* and *Tetragnatha hawaiensis* (Araneae, Tetragnathidae). Journal of Arachnology 19:210–214.

[eva12302-bib-0044] Croucher, P. J. P. , G. S. Oxford , A. Lam , N. Mody , and R. G. Gillespie 2012 Colonization history and population genetics of the color‐polymorphic Hawaiian happy‐face spider *Theridion grallator* (Araneae, Theridiidae). Evolution 66:2815–2833. 22946805 10.1111/j.1558-5646.2012.01653.x

[eva12302-bib-0045] Daly, H. V. , and K. N. Magnacca 2003 Insects of Hawaii: Hawaiian Hylaeus (Nesoprosopis) Bees (Hymenoptera: Apoidea), Vol. 17. University of Hawaii Press, Honolulu, 216 pp.

[eva12302-bib-0046] DeSalle, R. , and L. V. Giddings 1986 Discordance of nuclear and mitochondrial DNA phylogenies in Hawaiian *Drosophila* . Proceedings of the National Academy of Sciences of the USA 83:6902–6906. 3462736 10.1073/pnas.83.18.6902 PMC386618

[eva12302-bib-0047] Desalle, R. , L. V. Giddings , and K. Y. Kaneshiro 1986 Mitochondrial‐DNA variability in natural‐populations of Hawaiian *Drosophila*. 2. Genetic and phylogenetic relationships of natural populations of *Drosophila silvestris* and *Drosophila heteroneura* . Heredity 56:87–96. 3003013 10.1038/hdy.1986.12

[eva12302-bib-0048] Dunne, J. A. 2006 The network structure of food webs In Pascual M., and Dunne J. A., eds. Ecological Networks: Linking Structure to Dynamics in Food Webs, pp. 27–86. Oxford University Press, Oxford.

[eva12302-bib-0049] Eldredge, L. G. , and N. L. Evenhuis 2003 Hawaii's Biodiversity: A Detailed Assessment of the Numbers of Species in the Hawaiian Islands. Bishop Museum Press, Honolulu, pp 1–28.

[eva12302-bib-0050] Evenhuis, N. L. 2007 Barefoot on Lava: The Journals and Correspondence of Naturalist RCL Perkins in Hawai'i, 1892–1901, Vol. 7. Bishop Museum Press, Honolulu, 412 pp.

[eva12302-bib-0051] Fang, S. , R. Yukilevich , Y. Chen , D. A. Turissini , K. Zeng , I. A. Boussy , and C.‐I. Wu 2012 Incompatibility and competitive exclusion of genomic segments between sibling *Drosophila* species. PLoS Genetics 8:e1002795. 22761593 10.1371/journal.pgen.1002795 PMC3386244

[eva12302-bib-0052] Fukami, T. , H. J. E. Beaumont , X.‐X. Zhang , and P. B. Rainey 2007 Immigration history controls diversification in experimental adaptive radiation. Nature 446:436–439. 17377582 10.1038/nature05629

[eva12302-bib-0053] Funk, V. A. , and W. L. Wagner 1995 Biogeographic patterns in the Hawaiian Islands In Wagner W. L., and Funk V. A., eds. Hawaiian Biogeography Evolution on a Hot Spot Archipelago, pp. 379–419. Smithsonian Institution Press, Washington, DC.

[eva12302-bib-0054] Garb, J. E. , and R. G. Gillespie 2006 Island hopping across the central Pacific: mitochondrial DNA detects sequential colonization of the Austral Islands by crab spiders (Araneae: Thomisidae). Journal of Biogeography 33:201–220.

[eva12302-bib-0055] Garb, J. E. , and R. G. Gillespie 2009 Diversity despite dispersal: colonization history and phylogeography of Hawaiian crab spiders inferred from multilocus genetic data. Molecular Ecology 18:1746–1764. 19302468 10.1111/j.1365-294X.2009.04125.x

[eva12302-bib-0056] Gardner, R. C. 1976 Evolution and adaptive radiation in Lipochaeta (Compositae, Heliantheae) of the Hawaiian Islands. Systematic Botany 1:383–391.

[eva12302-bib-0200] Gertsch, W. J. 1973 The cavernicolous fauna of Hawaiian lava tubes, 3. Araneae (spiders). Pacific Insects 15:163–180.

[eva12302-bib-0057] Gillespie, R. G. 1991 Hawaiian spiders of the genus *Tetragnatha*: I. Spiny leg clade. Journal of Arachnology 19:174–209.

[eva12302-bib-0058] Gillespie, R. G. 1992 Hawaiian spiders of the genus *Tetragnatha* II. Species from natural areas of windward East Maui. Journal of Arachnology 20:1–17.

[eva12302-bib-0059] Gillespie, R. G. 1994 Hawaiian spiders of the genus *Tetragnatha*: III. *Tetragnatha acuta* clade. Journal of Arachnology 22:161–168.

[eva12302-bib-0060] Gillespie, R. G. 1999 Comparison of rates of speciation in web‐building and non‐web‐building groups within a Hawaiian spider radiation. Journal of Arachnology 27:79–85.

[eva12302-bib-0061] Gillespie, R. G. 2002a Colonization of remote oceanic islands of the Pacific: archipelagos as stepping stones? Journal of Biogeography 29:655–662.

[eva12302-bib-0062] Gillespie, R. G. 2002b Hawaiian spiders of the genus *Tetragnatha*: IV new, small species in the spiny leg clade. Journal of Arachnology 30:159–172.

[eva12302-bib-0063] Gillespie, R. G. 2003a Hawaiian spiders of the genus *Tetragnatha*: V. Elongate web‐builders from Oahu. Journal of Arachnology 31:8–19.

[eva12302-bib-0064] Gillespie, R. G. 2003b Marquesan spiders of the genus *Tetragnatha* . Journal of Arachnology 31:62–77.

[eva12302-bib-0065] Gillespie, R. G. 2003c Spiders of the genus *Tetragnatha* in the Society Islands. Journal of Arachnology 31:157–172.

[eva12302-bib-0066] Gillespie, R. G. 2004 Community assembly through adaptive radiation in Hawaiian spiders. Science 303:356–359. 14726588 10.1126/science.1091875

[eva12302-bib-0067] Gillespie, R. G. 2005a The ecology and evolution of Hawaiian spider communities. American Scientist 93:122–131.

[eva12302-bib-0068] Gillespie, R. G. 2005b Geographical context of speciation in a radiation of Hawaiian *Tetragnatha* spiders (Araneae, Tetragnathidae). Journal of Arachnology 33:313–322.

[eva12302-bib-0069] Gillespie, R. G. 2013 Adaptive radiation: convergence and non‐equilibrium. Current Biology 23:R71–R74. 23347943 10.1016/j.cub.2012.11.052

[eva12302-bib-0070] Gillespie, R. G. , and B. G. Baldwin 2009 Island biogeography of remote archipelagos: interplay between ecological and evolutionary processes In Losos J. B., and Ricklefs R. E., eds. The Theory of Island Biogeography Revisited, pp. 358–387. Princeton University Press, Princeton, NJ.

[eva12302-bib-0071] Gillespie, R. G. , and D. A. Clague 2009 Encyclopedia of Islands. University of California Press, Berkeley, CA, 1111 pp.

[eva12302-bib-0072] Gillespie, R. G. , and B. C. Emerson 2007 Adaptation under a microscope. Nature 446:386–387. 17377571 10.1038/446386a

[eva12302-bib-0073] Gillespie, R. G. , and G. S. Oxford 1998 Selection on the color polymorphism in Hawaiian happy‐face spiders: evidence from genetic structure and temporal fluctuations. Evolution 52:775–783. 10.1111/j.1558-5646.1998.tb03701.x 28565234

[eva12302-bib-0074] Gillespie, R. G. , and M. A. J. Rivera 2007 Free‐living spiders of the genus *Ariamnes* (Araneae, Theridiidae) in Hawaii. Journal of Arachnology 35:11–37.

[eva12302-bib-0075] Gillespie, R. G. , and B. E. Tabashnik 1989 What makes a happy face? Determinants of colour pattern in the Hawaiian happy face spider *Theridion grallator* (Araneae, Theridiidae). Heredity 62:355–363.

[eva12302-bib-0076] Gillespie, R. G. , H. B. Croom , and S. R. Palumbi 1994 Multiple origins of a spider radiation in Hawaii. Proceedings of the National Academy of Sciences of the USA 91:2290–2294. 8134390 10.1073/pnas.91.6.2290 PMC43356

[eva12302-bib-0077] Gillespie, R. G. , E. M. Claridge , and S. L. Goodacre 2008a Biogeography of the fauna of French Polynesia: diversification within and between a series of hot spot archipelagos. Philosophical Transactions of the Royal Society of London B: Biological Sciences 363:3335–3346. 18782725 10.1098/rstb.2008.0124 PMC2607382

[eva12302-bib-0078] Gillespie, R. G. , E. M. Claridge , and G. K. Roderick 2008b Biodiversity dynamics in isolated island communities: interaction between natural and human‐mediated processes. Molecular Ecology 17:45–57. 17727622 10.1111/j.1365-294X.2007.03466.x

[eva12302-bib-0079] Gillespie, R. G. , B. G. Baldwin , J. M. Waters , C. I. Fraser , R. Nikula , and G. K. Roderick 2012 Long‐distance dispersal: a framework for hypothesis testing. Trends in Ecology & Evolution 27:47–56. 22014977 10.1016/j.tree.2011.08.009

[eva12302-bib-0080] Givnish, T. J. , and K. J. Sytsma 1997 Molecular Evolution and Adaptive Radiation. Cambridge University Press, Cambridge, 621 pp.

[eva12302-bib-0081] Goodman, K. R. , S. C. Welter , and G. K. Roderick 2012 Genetic divergence is decoupled from ecological diversification in the Hawaiian *Nesosydne* planthoppers. Evolution 66:2798–2814. 22946804 10.1111/j.1558-5646.2012.01643.x

[eva12302-bib-0082] Graham, C. H. , A. C. Carnaval , C. D. Cadena , K. R. Zamudio , T. E. Roberts , J. L. Parra , C. M. McCain et al. 2014 The origin and maintenance of montane diversity: integrating evolutionary and ecological processes. Ecography 37:711–719.

[eva12302-bib-0083] Grant, P. R. 2000 RCL Perkins and evolutionary radiations on islands. Oikos 89:195–201.

[eva12302-bib-0300] Grant, P. R. , and B. R. Grant 2008 How and Why Species Multiply. Princeton University Press, Princeton & Oxford.

[eva12302-bib-0084] Grant, P. R. , B. R. Grant , and A. Abzhanov 2006 A developing paradigm for the development of bird beaks. Biological Journal of the Linnean Society 88:17–22.

[eva12302-bib-0085] Gressitt, J. L. 1982 Pacific‐Asian biogeography with examples from the Coleoptera. Entomologica Generalis 8:1–11.

[eva12302-bib-0086] Gruner, D. S. 2007 Geological age, ecosystem development, and local resource constraints on arthropod community structure in the Hawaiian Islands. Biological Journal of the Linnean Society 90:551–570.

[eva12302-bib-0087] Gruner, D. S. , D. A. Polhemus , Y. Basset , V. Novotny , S. E. Miller , and R. L. Kitching 2003 Arthropod assemblages across a long chronosequence in the Hawaiian Islands In Basset Y., Novotny V., Miller S. E., and Kitching R. L., eds. Arthropods of Tropical Forests: Spatio‐Temporal Dynamics and Resource Use in the Canopy, pp. 135–145. Cambridge University Press, Cambridge.

[eva12302-bib-0088] Hardy, D. 1965 Insects of Hawaii. Volume 12, Diptera: Cyclorrhapha II, Series Schizophora Section Acalypterae I. Family Drosophilidae. University of Hawaii Press, Honolulu.

[eva12302-bib-0089] Harmon, L. J. , J. B. Losos , T. J. Davies , R. G. Gillespie , J. L. Gittleman , W. B. Jennings , K. H. Kozak et al. 2010 Body size and shape rarely evolve in early bursts. Evolution 64:2385–2396. 20455932 10.1111/j.1558-5646.2010.01025.x

[eva12302-bib-0090] Harte, J. 2011 Maximum Entropy and Ecology: A Theory of Abundance, Distribution, and Energetics. Oxford University Press, Oxford.

[eva12302-bib-0091] Harte, J. , and E. A. Newman 2014 Maximum information entropy: a foundation for ecological theory. Trends in Ecology & Evolution 29:384–389. 24863182 10.1016/j.tree.2014.04.009

[eva12302-bib-0092] Harte, J. , A. B. Smith , and D. Storch 2009 Biodiversity scales from plots to biomes with a universal species‐area curve. Ecology Letters 12:789–797. 19486123 10.1111/j.1461-0248.2009.01328.x

[eva12302-bib-0093] Helenurm, K. , and F. R. Ganders 1985 Adaptive Radiation and Genetic Differentiation in Hawaiian Bidens. Evolution 39:753–765. 10.1111/j.1558-5646.1985.tb00417.x 28561363

[eva12302-bib-0094] Holmes, E. C. , and P. H. Harvey 1994 Spinning the web of life. Current Biology 9:841–843. 7820557 10.1016/s0960-9822(00)00188-3

[eva12302-bib-0400] Hormiga, G. 2002 *Orsonwelles*, a new genus of giant linyphiid spiders (Araneae) from the Hawaiian Islands. Invertebrate Systematics 16:369–448.

[eva12302-bib-0095] Hormiga, G. , M. Arnedo , and R. G. Gillespie 2003 Speciation on a conveyor belt: sequential colonization of the Hawaiian Islands by *Orsonwelles* spiders (Araneae, Linyphiidae). Systematic Biology 52:70–88. 12554442 10.1080/10635150390132786

[eva12302-bib-0096] Howarth, F. G. 1987 Evolutionary ecology of aeolian and subterranean habitats in Hawaii. Trends in Ecology & Evolution 2:220–223. 21227855 10.1016/0169-5347(87)90025-5

[eva12302-bib-0097] Howarth, F. G. , and B. H. Gagné 2012 Development of insect conservation in Hawai'i Insect Conservation: Past, Present and Prospects. Springer, Berlin.

[eva12302-bib-0098] Hubbell, S. P. 2001 The Unified Neutral Theory of Biodiversity and Biogeography (MPB‐32), Vol. 32. Princeton University Press, Princeton, NJ.

[eva12302-bib-0099] Huffaker, C. B. 1958 Experimental studies on predation: dispersion factors and predator‐prey oscillations. Hilgardia 27:343–383.

[eva12302-bib-0100] Hutter, C. R. , J. M. Guayasamin , and J. J. Wiens 2013 Explaining Andean megadiversity: the evolutionary and ecological causes of glassfrog elevational richness patterns. Ecology Letters 16:1135–1144. 23802805 10.1111/ele.12148

[eva12302-bib-0101] Iturralde‐Vinent, M. A. , and R. D. E. MacPhee 1999 Paleogeography of the Caribbean region: implications for Cenzoic biogeography. Bulletin of the American Museum of Natural History 238:1–95.

[eva12302-bib-0102] Johnson, M. T. J. , and J. R. Stinchcombe 2007 An emerging synthesis between community ecology and evolutionary biology. Trends in Ecology and Evolution 22:250–257. 17296244 10.1016/j.tree.2007.01.014

[eva12302-bib-0103] Joy, D. A. , and J. E. Conn 2001 Molecular and morphological phylogenetic analysis of an insular radiatin in Pacific black flies (*Simulium*). Systematic Biology 50:18–38. 12116592

[eva12302-bib-0104] Kambysellis, M. P. , K.‐F. Ho , E. M. Craddock , F. Piano , M. Parisi , and J. Cohen 1995 Pattern of ecological shifts in the diversification of Hawaiian *Drosophila* inferred from a molecular phylogeny. Current Biology 5:1129–1139. 8548285 10.1016/s0960-9822(95)00229-6

[eva12302-bib-0105] Kaneshiro, K. Y. 1997 R.C.L. Perkins’ legacy to evolutionary research on Hawaiian Drosophilidae, Diptera. Pacific Science 51:450–461.

[eva12302-bib-0106] Kolbe, J. J. , R. E. Glor , L. R. Schettino , A. C. Lara , A. Larson , and J. B. Losos 2007 Multiple sources, admixture, and genetic variation in introduced *Anolis* lizard populations. Conservation Biology 21:1612–1625. 18173485 10.1111/j.1523-1739.2007.00826.x

[eva12302-bib-0107] Krushelnycky, P. D. , and R. G. Gillespie 2008 Compositional and functional stability of arthropod communities in the face of ant invasions. Ecological Applications 18:1547–1562. 18767628 10.1890/07-1293.1

[eva12302-bib-0108] Krushelnycky, P. D. , and R. G. Gillespie 2010 Correlates of vulnerability among arthropod species threatened by invasive ants. Biodiversity and Conservation 19:1971–1988.

[eva12302-bib-0109] Langerhans, R. B. , J. H. Knouft , and J. B. Losos 2006 Shared and unique features of diversification in Greater Antillean *Anolis* ecomorphs. Evolution 60:362–369. 16610326

[eva12302-bib-0500] Lehtinen, P. T. , and Y. M. Marusik 2008 A redefinition of *Misumenops* F. O. Pickard‐Cambridge, 1900 (Araneae, Thomisidae) and review of the New World species. Bulletin of the British Arachnological Society 14:173–198.

[eva12302-bib-0110] Lessard, J.‐P. , J. Belmaker , J. A. Myers , J. M. Chase , and C. Rahbek 2012 Inferring local ecological processes amid species pool influences. Trends in Ecology & Evolution 27:600–607. 22877982 10.1016/j.tree.2012.07.006

[eva12302-bib-0111] Liebherr, J. K. , and E. C. Zimmerman 2000 Coleoptera: Carabidae: Part 1: Introduction and Tribe Platynini. Volume 16, Insects of Hawaii. University of Hawai'i Press, Honolulu.

[eva12302-bib-0112] Loeuille, N. , and M. Loreau 2005 Evolutionary emergence of size‐structured food webs. Proceedings of the National Academy of Sciences of the USA 102:5761–5766. 15824324 10.1073/pnas.0408424102 PMC556288

[eva12302-bib-0113] Loh, Y.‐H. E. , E. Bezault , F. M. Muenzel , R. B. Roberts , R. Swofford , M. Barluenga , C. E. Kidd et al. 2013 Origins of shared genetic variation in African cichlids. Molecular Biology and Evolution 30:906–917. 23275489 10.1093/molbev/mss326 PMC3603313

[eva12302-bib-0114] Losos, J. B. 2009 Lizards in an Evolutionary Tree: Ecology and Adaptive Radiation of Anoles. University of California Press, Berkeley, CA.

[eva12302-bib-0115] Losos, J. B. 2010 Adaptive radiation, ecological opportunity, and evolutionary determinism. The American Naturalist 175:623–639. 10.1086/652433 20412015

[eva12302-bib-0116] Losos, J. B. , and R. E. Ricklefs 2009 The Theory of Island Biogeography Revisited. Princeton University Press, Princeton, NJ.

[eva12302-bib-0117] Lowrey, T. K. , and D. J. Crawford 1985 Allozyme divergence and evolution in *Tetramolopium* (Compositae, Asteraceae) on the Hawaiian Islands. Systematic Botany 10:64–72.

[eva12302-bib-0118] Lund, J. W. 1999 Historical impacts of geothermal resources on the people of North America In Cataldi R., Hodgson S., and Lund J. W., eds. Stories from a Heated Earth: Our Geothermal Heritage, Vol. 19, pp. 451 Geothermal Resources Council (GRC) and International Geothermal Association (IGA), Sacramento, CA.

[eva12302-bib-0600] MacArthur, R. H. , and E. O. Wilson 1967 The Theory of Island Biogeography. Princeton University Press, Princeton, NJ.

[eva12302-bib-0119] May, R. M. 2001 Stability and Complexity in Model Ecosystems, 2nd edn Princeton University Press, Princeton, NJ.

[eva12302-bib-0120] McCormack, J. E. , H. Huang , and L. L. Knowles 2009 Sky islands. Encyclopedia of Islands 4:841–843.

[eva12302-bib-0121] McGill, B. J. 2010 Towards a unification of unified theories of biodiversity. Ecology Letters 13:627–642. 20337695 10.1111/j.1461-0248.2010.01449.x

[eva12302-bib-0122] Melián, C. J. , O. Seehausen , V. M. Eguíluz , M. A. Fortuna , and K. Deiner 2015 Diversification and biodiversity dynamics of hot and cold spots. Ecography 38:393–401.

[eva12302-bib-0123] Mendelson, T. C. , and K. L. Shaw 2005 Sexual behaviour: rapid speciation in an arthropod. Nature 433:375–376. 15674280 10.1038/433375a

[eva12302-bib-0124] Meyer, J. R. , and R. Kassen 2007 The effects of competition and predation on diversification in a model adaptive radiation. Nature 446:432–435. 17377581 10.1038/nature05599

[eva12302-bib-0125] Mittelbach, G. G. , and D. W. Schemske 2015 Ecological and evolutionary perspectives on community assembly. Trends in Ecology & Evolution 30:241–247. 25804867 10.1016/j.tree.2015.02.008

[eva12302-bib-0126] Mueller‐Dombois, D. 1987 Forest dynamics in Hawaii. Trends in Ecology & Evolution 2:216–220. 21227854 10.1016/0169-5347(87)90024-3

[eva12302-bib-0127] Muschick, M. , M. Barluenga , W. Salzburger , and A. Meyer 2011 Adaptive phenotypic plasticity in the Midas cichlid fish pharyngeal jaw and its relevance in adaptive radiation. BMC Evolutionary Biology 11:116. 21529367 10.1186/1471-2148-11-116 PMC3103464

[eva12302-bib-0128] Muschick, M. , A. Indermaur , and W. Salzburger 2012 Convergent evolution within an adaptive radiation of cichlid fishes. Current Biology 22:2362–2368. 23159601 10.1016/j.cub.2012.10.048

[eva12302-bib-0129] Nishida, G. M. 2002 Hawaiian Terrestrial Arthropod Checklist. Hawaii Biological Survey, Bishop Museum, Honolulu.

[eva12302-bib-0130] O'Grady, P. M. , R. T. Lapoint , J. Bonacum , J. Lasola , E. Owen , Y. Wu , and R. DeSalle 2011 Phylogenetic and ecological relationships of the Hawaiian *Drosophila* inferred by mitochondrial DNA analysis. Molecular Phylogenetics and Evolution 58:244–256. 21144904 10.1016/j.ympev.2010.11.022

[eva12302-bib-0131] O'Quin, K. E. , A. R. Smith , A. Sharma , and K. L. Carleton 2011 New evidence for the role of heterochrony in the repeated evolution of cichlid opsin expression. Evolution and Development 13:193–203. 21410875 10.1111/j.1525-142X.2011.00469.x

[eva12302-bib-0133] Oxford, G. S. , and R. G. Gillespie 1996a Genetics of a colour polymorphism in *Theridion grallator* (Araneae: Theridiidae), the Hawaiian happy‐face spider, from Greater Maui. Heredity 76:238–248.

[eva12302-bib-0134] Oxford, G. S. , and R. G. Gillespie 1996b Quantum shifts in the genetic control of a colour polymorphism in *Theridion grallator* (Araneae: Theridiidae), the Hawaiian happy‐face spider. Heredity 76:249–256.

[eva12302-bib-0132] Oxford, G. S. , and R. G. Gillespie 1996c The effects of genetic background on the island‐specific control of a colour polymorphism in *Theridion grallator* (Araneae: Theridiidae), the Hawaiian happy‐face spider. Heredity 76:257–266.

[eva12302-bib-0135] Oxford, G. S. , and R. G. Gillespie 2001 Portraits of evolution: studies of coloration in Hawaiian spiders. BioScience 51:521–528.

[eva12302-bib-0136] Page, R. D. M. 2013 Biodiversity informatics in charts. *iPhylo*, http://iphylo.blogspot.co.uk/2013/09/biodiversity-informatics-in-charts.html (accessed on 11 September 2015).

[eva12302-bib-0137] Paine, R. T. 1966 Food web complexity and species diversity. The American Naturalist 100:65–75.

[eva12302-bib-0138] Palkovacs, E. P. , and A. P. Hendry 2010 Eco‐evolutionary dynamics: intertwining ecological and evolutionary processes in contemporary time. F1000 Biology Reports 2:1–5. 20948823 10.3410/B2-1 PMC2948349

[eva12302-bib-0139] Palmer, M. W. 1994 Variation in species richness: towards a unification of hypotheses. Folia Geobotanica et Phytotaxonomica 29:511–530.

[eva12302-bib-0140] Perkins, R. C. L. 1903 Fauna Hawaiiensis, or the Zoology of the Sandwich (Hawaiian) Isles: Being Results of the Explorations Instituted by the Joint Committee Appointed by the Royal Society of London for Promoting Natural Knowledge and the British Association for the Advancement of Science, and Carried on with the Assistance of Those Bodies and of the Trustees of the Bernice Pauahi Bishop Museum at Honolulu. Cambridge University Press, Cambridge.

[eva12302-bib-0141] Perkins, R. C. L. 1913 Introduction (to Fauna Hawaiiensis) Fauna Hawaiiensis 1. Cambridge University Press, Cambridge.

[eva12302-bib-0142] Pfennig, D. W. , M. A. Wund , E. C. Snell‐Rood , T. Cruickshank , C. D. Schlichting , and A. P. Moczek 2010 Phenotypic plasticity's impacts on diversification and speciation. Trends in Ecology and Evolution 25:459–467. 20557976 10.1016/j.tree.2010.05.006

[eva12302-bib-0143] Pianka, E. R. 1966 Latitudinal gradients in species diversity: a review of concepts. The American Naturalist 100:33–46.

[eva12302-bib-0144] Porembski, S. 2009 Inselbergs In Gillespie R. G., and Clague D. A., eds. Encyclopedia of Islands, pp. 466–469. University of California Press, Berkeley, CA.

[eva12302-bib-0145] Price, J. P. , and D. A. Clague 2002 How old is the Hawaiian biota? Geology and phylogeny suggest recent divergence. Proceedings of the Royal Society of London B: Biological Sciences 269:2429–2435. 10.1098/rspb.2002.2175 PMC1691179 12495485

[eva12302-bib-0146] Price, T. D. , A. Qvarnstrom , and D. E. Irwin 2003 The role of phenotypic plasticity in driving genetic evolution. Proceedings of the Royal Society of London B: Biological Sciences 270:1433–1440. 10.1098/rspb.2003.2372 PMC1691402 12965006

[eva12302-bib-0700] Prószyn'ski, J. 2002 Remarks on Salticidae (Aranei) from Hawaii, with description of *Havaika* gen.n. Arthropoda Selecta 10:225–241.

[eva12302-bib-0800] Prószyn'ski, J. 2008 A survey of Havaika (Aranei: Salticidae), an endemic genus from Hawaii, including descriptions of new species. Arthropoda Selecta 16:195–213.

[eva12302-bib-0147] Rabosky, D. L. , and R. E. Glor 2010 Equilibrium speciation dynamics in a model adaptive radiation of island lizards. Proceedings of the National Academy of Sciences of the USA 107:22178–22183. 21135239 10.1073/pnas.1007606107 PMC3009809

[eva12302-bib-0148] Ritchie, M. G. 2007 Sexual selection and speciation. Annual Review of Ecology, Evolution, and Systematics 38:79–102.

[eva12302-bib-0149] Rius, M. , and J. A. Darling 2014 How important is intraspecific genetic admixture to the success of colonising populations? Trends in Ecology & Evolution 29:233–242. 24636862 10.1016/j.tree.2014.02.003

[eva12302-bib-0150] Roderick, G. K. , and R. G. Gillespie 1998 Speciation and phylogeography of Hawaiian terrestrial arthropods. Molecular Ecology 7:519–531. 9628003 10.1046/j.1365-294x.1998.00309.x

[eva12302-bib-0151] Roderick, G. K. , P. J. P. Croucher , A. Vandergast , and R. G. Gillespie 2012 Species differentiation on a dynamic landscape: shifts in metapopulation and genetic structure using the chronology of the Hawaiian archipelago. Evolutionary Biology 32:192–206. 22707805 10.1007/s11692-012-9184-5 PMC3364410

[eva12302-bib-0152] Roesti, M. , S. Gavrilets , A. P. Hendry , W. Salzburger , and D. Berner 2014 The genomic signature of parallel adaptation from shared genetic variation. Molecular Ecology 23:3944–3956. 24635356 10.1111/mec.12720 PMC4122612

[eva12302-bib-0153] Rohde, K. 1992 Latitudinal gradients in species diversity: the search for the primary cause. Oikos 65:514–527.

[eva12302-bib-0154] Romanuk, T. N. , Y. Zhou , U. Brose , E. L. Berlow , R. J. Williams , and N. D. Martinez 2009 Predicting invasion success in complex ecological networks. Philosophical Transactions of the Royal Society of London B: Biological Sciences 364:1743–1754. 19451125 10.1098/rstb.2008.0286 PMC2685429

[eva12302-bib-0155] Rominger, A. J. , K. R. Goodman , J. Y. Lim , F. S. Valdovinos , E. Armstrong , G. M. Bennett , M. S. Brewer et al. 2015 Community assembly on isolated islands: macroecology meets evolution. Global Ecology and Biogeography doi: 10.1111/geb.12341.

[eva12302-bib-0156] Rull, V. 2009 Pantepui In Gillespie R. G., and Clague D. A., eds. Encyclopedia of Islands, pp. 717–720. University of California Press, Berkeley, CA.

[eva12302-bib-0157] Sakai, A. K. , W. L. Wagner , D. M. Ferguson , and D. R. Herbst 1995 Biogeographical and ecological correlates of dioecy in the Hawaiian flora. Ecology 76:2530–2543.

[eva12302-bib-0900] Schluter, D. 2000 The Ecology of Adaptive Radiation. Oxford University Press, Oxford.

[eva12302-bib-0158] Schluter, D. 2003 Frequency dependent natural selection during character displacement in sticklebacks. Evolution 57:1142–1150. 12836830 10.1111/j.0014-3820.2003.tb00323.x

[eva12302-bib-0159] Schluter, D. 2009 Evidence for ecological speciation and its alternative. Science 323:737–741. 19197053 10.1126/science.1160006

[eva12302-bib-0160] Schluter, D. , and L. Nagel 1995 Parallel speciation by natural selection. The American Naturalist 146:292–301.

[eva12302-bib-0161] Schoville, S. D. , G. K. Roderick , and D. H. Kavanaugh 2012 Testing the “Pleistocene species pump” in alpine habitats: lineage diversification of flightless ground beetles (Coleoptera: Carabidae: *Nebria*) in relation to altitudinal zonation. Biological Journal of the Linnean Society 107:95–111.

[eva12302-bib-0162] Seehausen, O. 2009 Ecology: speciation affects ecosystems. Nature 458:1122–1123. 19407790 10.1038/4581122a

[eva12302-bib-0163] Seehausen, O. L. E. , G. Takimoto , D. Roy , and J. Jokela 2008 Speciation reversal and biodiversity dynamics with hybridization in changing environments. Molecular Ecology 17:30–44. 18034800 10.1111/j.1365-294X.2007.03529.x

[eva12302-bib-0164] Severin, T. 1997 The Spice Islands Voyage: The Quest for Alfred Wallace, the Man Who Shared Darwin's Discovery of Evolution. Carroll & Graf Publishers, New York.

[eva12302-bib-0165] Sibley, S. G. , and J. E. Ahlquist 1982 The relationships of the Hawaiian honeycreepers (Drepaninini) as indicated by DNA‐DNA hybridization. The Auk 99:130–140.

[eva12302-bib-0166] Simberloff, D. S. , and E. O. Wilson 1970 Experimental zoogeography of islands. A two‐year record of colonization. Ecology 51:934–937.

[eva12302-bib-0167] Simon, E. 1900 In D Sharp, ed. Fauna Hawaiiensis, Vol. 2, pp. 443–519, pls. 415–419. Arachnida. Cambridge University Press, Cambridge.

[eva12302-bib-0168] Simon, C. 1987 Hawaiian evolutionary biology: an introduction. Trends in Ecology & Evolution 2:175–178. 21227845 10.1016/0169-5347(87)90015-2

[eva12302-bib-0169] Steiner, C. E. , Z. T. Long , J. A. Krumins , and P. J. Morin 2006 Population and community resilience in multitrophic communities. Ecology 87:996–1007. 16676543 10.1890/0012-9658(2006)87[996:pacrim]2.0.co;2

[eva12302-bib-0170] Suman, T. W. 1970 Spiders of the family Thomisidae in Hawaii, Pacific Insects. 12:773–864.

[eva12302-bib-0171] Sutherst, R. W. , G. F. Maywald , and A. S. Bourne 2007 Including species interactions in risk assessments for global change. Global Change Biology 13:1843–1859.

[eva12302-bib-0172] Triantaphyllidis, C. D. , and R. H. Richardson 1982 DNA/DNA hybridization studies among six endemic Hawaiian *Drosophila* species. Genetica 57:225–229.

[eva12302-bib-0173] Triantis, K. A. , and S. A. Bhagwat 2011 Applied island biogeography In Ladle R. J., and Whittaker R. J., eds. Conservation Biogeography, pp. 190–223. Wiley‐Blackwell, Oxford.

[eva12302-bib-0174] Tylianakis, J. M. , E. Laliberté , A. Nielsen , and J. Bascompte 2010 Conservation of species interaction networks. Biological Conservation 143:2270–2279.

[eva12302-bib-0175] Vandergast, A. G. , R. G. Gillespie , and G. K. Roderick 2004 Influence of volcanic activity on the population genetic structure of Hawaiian *Tetragnatha* spiders: fragmentation, rapid population growth and the potential for accelerated evolution. Molecular Ecology 13:1729–1743. 15189199 10.1111/j.1365-294X.2004.02179.x

[eva12302-bib-0176] Vanderwerf, E. A. 2012 Evolution of nesting height in an endangered Hawaiian forest bird in response to a non‐native predator. Conservation Biology 26:905–911. 22830652 10.1111/j.1523-1739.2012.01877.x

[eva12302-bib-0177] Vellend, M. 2010 Conceptual synthesis in community ecology. The Quarterly Review of Biology 85:183–206. 20565040 10.1086/652373

[eva12302-bib-0178] Vitousek, P. M. 2004 Nutrient Cycling and Limitation: Hawai'i as a Model System. Princeton University Press, Princeton, NJ.

[eva12302-bib-0179] Vitousek, P. M. , L. L. Loope , and C. P. Stone 1987 Introduced species in Hawaii. Biological effects and opportunities for ecological research. Trends in Ecology & Evolution 2:224–227. 21227856 10.1016/0169-5347(87)90026-7

[eva12302-bib-0180] Vitousek, P. M. , D. R. Turner , and K. Kitayama 1995 Foliar nutrients during long‐term soil development in Hawaiian montane rain forest. Ecology 76:712–720.

[eva12302-bib-0181] Wagner W. L., and Funk V. A., eds. 1995 Hawaiian Biogeography: Evolution on a Hot Spot Archipelago. Smithsonian Institution Press, Washington, DC.

[eva12302-bib-0182] Wagner, C. E. , L. J. Harmon , and O. Seehausen 2014 Cichlid species‐area relationships are shaped by adaptive radiations that scale with area. Ecology Letters 17:583–592. 24602171 10.1111/ele.12260

[eva12302-bib-0183] Wardle, D. A. 2006 The influence of biotic interactions on soil biodiversity. Ecology Letters 9:870–886. 16796577 10.1111/j.1461-0248.2006.00931.x

[eva12302-bib-0184] Wardle, D. A. , L. R. Walker , and R. D. Bardgett 2004 Ecosystem properties and forest decline in contrasting long‐term chronosequences. Science 305:509–513. 15205475 10.1126/science.1098778

[eva12302-bib-1000] Warren, B. H. , D. Simberloff , R. E. Ricklefs , R. Aguilée , F. L. Condamine , D. Gravel , H. Morlon , et al. 2015 Islands as model systems in ecology and evolution: prospects fifty years after MacArthur‐Wilson. Ecology Letters 18:200–217. 25560682 10.1111/ele.12398

[eva12302-bib-0185] Weir, J. T. , and D. Schluter 2004 Ice sheets promote speciation in boreal birds. Proceedings of the Royal Society of London B: Biological Sciences 271:1881–1887. 10.1098/rspb.2004.2803 PMC1691815 15347509

[eva12302-bib-0186] West‐Eberhard, M. J. 2003 Developmental Plasticity and Evolution. Oxford University Press, Oxford.

[eva12302-bib-0187] West‐Eberhard, M. J. 2005 Developmental plasticity and the origin of species differences. Proceedings of the National Academy of Sciences of the USA 102(Suppl 1):6543–6549. 15851679 10.1073/pnas.0501844102 PMC1131862

[eva12302-bib-0188] White, E. , S. Ernest , A. Kerkhoff , and B. Enquist 2007 Relationships between body size and abundance in ecology. Trends in Ecology and Evolution 22:323–330. 17399851 10.1016/j.tree.2007.03.007

[eva12302-bib-0189] Wiens, J. J. , R. A. Pyron , and D. S. Moen 2011 Phylogenetic origins of local‐scale diversity patterns and the causes of Amazonian megadiversity. Ecology Letters 14:643–652. 21535341 10.1111/j.1461-0248.2011.01625.x

[eva12302-bib-0190] Wilbur, H. M. 1997 Experimental ecology of food webs: complex systems in temporary ponds. Ecology 78:2279–2302.

[eva12302-bib-0191] Williams, R. J. 2010 Simple MaxEnt models explain foodweb degree distributions. Theoretical Ecology 3:45–52.

[eva12302-bib-0192] Williams, R. J. , and N. D. Martinez 2004 Stabilization of chaotic and non‐permanent food web dynamics. European Physical Journal B: Condensed Matter and Complex Systems 38:297–303.

[eva12302-bib-0193] Williams, R. J. , and N. D. Martinez 2008 Success and its limits among structural models of complex food webs. Journal of Animal Ecology 77:512–519. 18284474 10.1111/j.1365-2656.2008.01362.x

[eva12302-bib-0194] Williamson, M. 1996 Biological Invasions. Chapman & Hall, London, UK.

[eva12302-bib-0195] Wilson, J. T. 1963 A possible origin of the Hawaiian Islands. Canadian Journal of Physics 41:863–870.

[eva12302-bib-0196] Witter, M. S. , and G. D. Carr 1988 Adaptive radiation and genetic differentiation in the Hawaiian silversword alliance (Compositae: Madiinae). Evolution 42:1278–1287. 10.1111/j.1558-5646.1988.tb04187.x 28581078

[eva12302-bib-0197] Zimmerman, E. C. 1948 Introduction. Vol. 1, Insects of Hawaii. University of Hawaii Press, Honolulu.

[eva12302-bib-0198] Zimmerman, E. C. 1958 300 species of *Drosophila*? – A challenge to geneticists and evolutionists. Evolution 12:557–558.

